# RNA metagenomic profiling of mosquito viromes associated with Vector-Borne diseases in Quebec, Canada

**DOI:** 10.1371/journal.pone.0350663

**Published:** 2026-06-05

**Authors:** Ines Levade, Benjamin Delisle, Éric Fournier, Christian Therrien

**Affiliations:** Laboratoire de santé publique du Québec, Institut de santé publique du Québec, Sainte-Anne-de-Bellevue, Québec, Canada; University of Ibadan Faculty of Veterinary Medicine, NIGERIA

## Abstract

Mosquitoes harbor diverse viral communities, including both medically important arboviruses and insect-specific viruses, yet the viromes of mosquito populations in northern temperate regions remains poorly characterized. In this study, we used metagenomic sequencing to analyse pools of archived mosquito samples from Québec, Canada representing multiple species previously identified as arbovirus carriers. Our analyses identified 60 viral species, including three arboviruses, several insect-specific viruses, and multiple dual-host non-pathogenic viruses, revealing the rich viral diversity present in these mosquito populations. Phylogenetic analysis of complete viral genomes demonstrated genetic relationships with viruses reported from diverse geographic regions. We describe, a newly proposed bipartite *Culex tombus-*like virus and report the complete resolution of thirty-five viral genomic sequences. These results highlight the utility of metagenomic approaches for comprehensive characterization of the mosquito virome and underscore their potential to enhance surveillance of emerging arboviruses, including West Nile virus, in Québec and similar northern ecosystems.

## Introduction

Recent advances in high-throughput sequencing and metagenomic approaches have significantly expanded our understanding of the mosquito virome, revealing a complex and diverse assemblage of viruses associated with mosquito populations worldwide [1–3]. These viral communities include both vertebrate-infecting arboviruses and a wide range of insect-specific viruses (ISVs) that replicate exclusively in invertebrate hosts. Metagenomic analyses have shown that mosquito viromes can vary widely across mosquito species, ecological settings, and geographic regions, reflecting the influence of environmental conditions, host ecology, and vector population dynamics [4–8]. Characterizing these viral communities is therefore important for understanding virus diversity and the ecological processes shaping virus circulation in mosquito populations.

In addition to medically important arboviruses, mosquito viromes often contain numerous ISVs belonging to diverse viral families. Although these viruses do not infect vertebrate hosts, growing evidence suggests that they may influence mosquito biology, immunity, and vector competence through interactions with pathogenic viruses [2,9,10]. Consequently, profiling mosquito viromes provides valuable insights not only into viral diversity but also into potential virus–virus and virus–vector interactions that may affect the transmission dynamics of arboviruses. Metagenomic sequencing has become a powerful tool for detecting both known and previously undescribed viruses in mosquito samples, enabling unbiased characterization of viral communities and improving surveillance of emerging pathogens.

Despite the increasing number of mosquito virome studies conducted globally, significant geographic gaps remain, particularly in northern temperate regions. In Canada, and especially in the province of Québec, research on mosquito-associated viral communities has primarily focused on the surveillance of medically important arboviruses such as West Nile virus, with limited information available on the broader mosquito virome [11,12]. Expanding virome characterization in these regions is therefore essential for improving our understanding of virus diversity in local mosquito populations and for strengthening surveillance frameworks aimed at detecting emerging vector-borne viruses.

Climate change is expected to modify temperature and precipitation patterns in ways that favour mosquito proliferation as mosquitoes are ectothermic and depend on aquatic habitats for larval development. In North America, West Nile virus (WNV) was first detected in 1999 in *Culex pipiens* mosquitoes from New York City [13] and spread to Québec by 2002, particularly along the St. Lawrence River Valley [14]. Between 2003–2018, 34% of all mosquito traps tested positive at least once during this time course across twelve health regions. The infection rate in *Culex pipiens* varied markedly across regions, ranging from 0.4 to 26.5 per 1,000 mosquitoes. [12]. Other arboviruses have also been detected, including Eastern equine encephalitis virus (EEEV) in *Culiseta melanura* near Montréal and Snowshoe Hare virus (SSHV) in *Ochlerotatus stimulans* in the Abitibi-Témiscamingue region [15,16]. Major peaks in human WNV cases occurred in 2012, 2018 and 2025,and the first human EEEV cases were reported in 2024 [16,17]. Together, these trends suggest that arboviral disease risk in Québec may increase, underscoring the need for strengthened surveillance to protect public health.

Highly sensitive RT-PCR assays remain widely used in arbovirus surveillance but are limited detecting viruses with known genomic sequences. In contrast, metagenomic sequencing of mosquito homogenates enables untargeted detection of microorganisms within the mosquito microbiota, facilitating the discovery of novel or divergent viruses and the recovery of complete viral genomes for phylogenetic and evolutionary analysis. Genomic data can also reveal mutations linked to virulence or vector adaptation. In addition, growing evidence shows that interactions within the mosquito microbiota can influence arbovirus transmission; for example, ISVs may inhibit replication of dual-host flaviviruses such as WNV, and the bacterial endosymbiont *Wolbachia* can similarly interfere with arbovirus replication in mosquitoes [18–20].

In this study, we aimed to characterize the virome of mosquito populations in Québec, Canada, using pools of mosquitoes representing multiple species previously identified as carriers of arboviruses. By applying unbiased RNA metagenomic sequencing, we sought to identify both known arboviruses, such as West Nile virus, and insect-specific viruses that comprise the broader mosquito virome. As an inherent consequence of unbiased total RNA sequencing, our dataset also captures messenger and ribosomal RNA from the mosquito-associated microbiota, including endosymbiotic bacteria such as *Wolbachia*, whose presence and transcriptional activity in mosquitoes are well documented and have known implications for arboviral susceptibility and transmission [18–20]. This work represents the first systematic virome analysis conducted in Québec and contributes to filling a major knowledge gap regarding virus diversity and circulation in northern temperate mosquito populations. Ultimately, our study provides baseline data that can inform vector surveillance programs and enhance preparedness for emerging mosquito-borne viruses in Canada.

## Materials and methods

### Mosquito collection and arbovirus screening

Over 44,000 mosquito pools were collected between 2003 and 2016 as part of the Québec provincial entomological surveillance program. Mosquitoes were collected overnight using CDC light traps from GDG Environnement (Kersia, Canada). Mosquitoes were identified to species level using morphological identification keys and subsequently grouped into pools accordingly. The collection of mosquito species known to be competent vectors of medically important arboviruses were prioritized in the established surveillance program for arboviruses (*Culex sp, Culiseta sp, Ochlerotataus, Aedes sp*). All samples were stored at -70^o^C until further processing.

**Fig 1:**
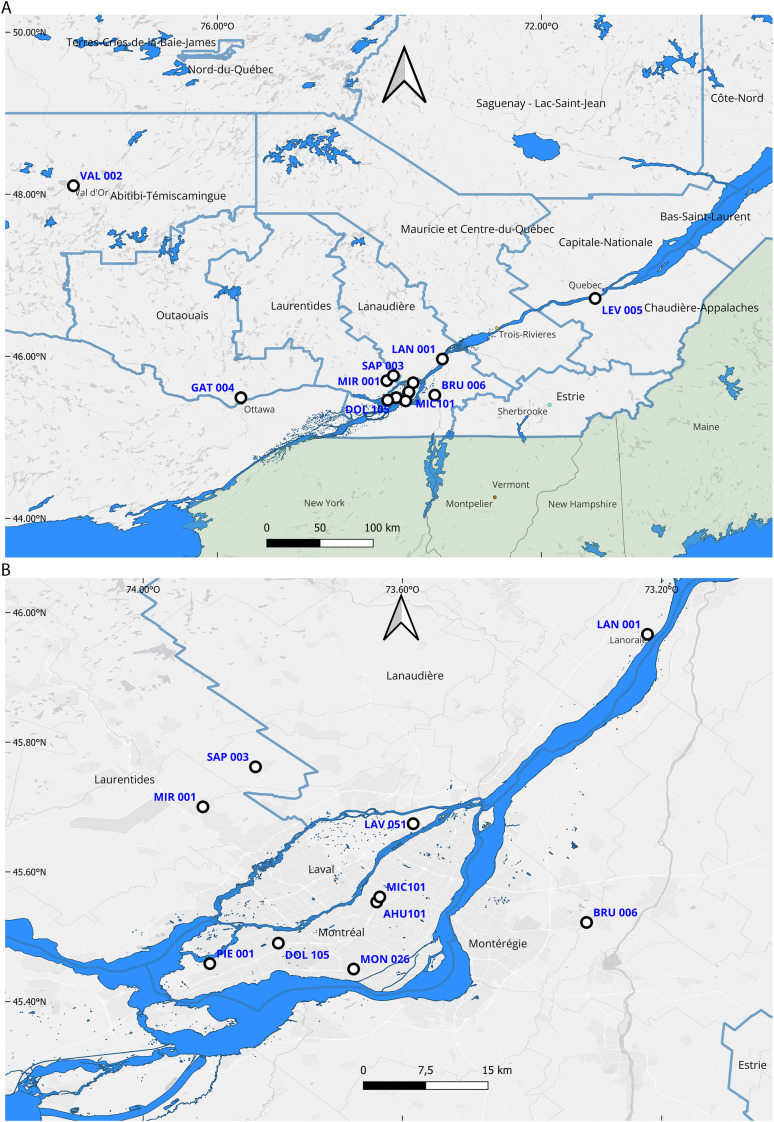
Geographic location of mosquito samples analysed in this study. The location of thirteen sampling sites is marked with black circles **(A)**. The trapping sites located in the greater Montreal region are shown in **(B)**.

In total, sixteen pools of mosquitoes were selected for microbial metagenomic assessment including eleven pools of *Culex pipiens* positive for WNV, two pools of *Ochlerotatus stimulans* positive for SSHV and two pools of *Culiseta melanura* positive for EEEV. No other arbovirus-infected *Culiseta* or *Ochlerotatus* species were available, preventing a thorough comparison of viromes across species. We also selected one *Culex pipiens* sample negative for WNV. The trapping sites, vector species and the number of mosquitoes per pool are presented in **Table 1**. The samples were collected from 13 stations located in nine Health units (**Fig 1A**). Most sampling stations were concentrated in the southern part of the Greater Montreal area (**Fig 1B**). Three additional stations were located elsewhere in Québec: one in the Abitibi-Témiscamingue Health Unit (northwest), one in the Capitale-Nationale Health Unit (east), and one in the Outaouais Health Unit (west) (Fig 1A). The map was created with QGIS Desktop 3.38.0. using trapping site coordinates from GDG environnement, the Québec provincial hydrographic and Health unit regions datasets (GRQ, MNRF Hydro, and RSS QC-Territoires_RSS_2022) from the Québec Open Data Portal under the Open Government Licence – Québec, the Cartographic Boundary Files (USA states_2018) from the U.S. federal government and the ESRI Gray Light basemap.

**Table 1:** Mosquito species analysed in this study and trapping site location.

Samples	Mosquito species	Collection Year	Trap ID	Health unit	No mosquito per pool
13896CPR1	*Culex pipiens*	2005	GAT004	Outaouais	36
13386CPR1	*Culex pipiens*	2005	SAP003	Laurentide	1
12739CPR3	*Culex pipiens*	2005	LAV051	Laval	50
12867CPR2	*Culex pipiens*	2005	MIR001	Laurentides	30
VN4593CPR1	*Culex pipiens*	2014	AHU101	Montréal	15
VN4608CPR1	*Culex pipiens*	2014	DOL105	Montréal	23
VN6387CPR1	*Culex pipiens*	2015	MIC101	Montréal	32
VN6325CPR1	*Culex pipiens*	2015	BRU006	Montérégie	14
VN6477CPR1	*Culex pipiens*	2015	PIE001	Montréal	5
VN6307CPR1	*Culex pipiens*	2015	MON026	Montréal	3
VN6831CPR1	*Culex pipiens*	2016	MIC101	Montréal	50
VN6800CPR1	*Culex pipiens*	2016	LEV005	Chaudières-appalaches	1
VN6827MEL1	*Culiseta melanura*	2016	LAN001	Lanaudière	28
VN7173MEL1	*Culiseta melanura*	2016	LAN001	Lanaudière	30
VN6966SMG1	*Ochlerotatus stimulans*	2016	VAL002	Abitibi-Témiscaminque	7
VN6711SMG1	*Ochlerotatus stimulans*	2016	VAL002	Abitibi-Témiscaminque	20

### RNA extraction, library preparation and sequencing

The mosquito pools were homogenized with a mixer Mill 300 (Qiagen, Montreal, Canada) at 30 Hz for 2 minutes and centrifuged at 13,500 RPM for 4 minutes at 2–8 °C. Total RNA was extracted using RNeasy 96 kit (Qiagen, Montreal, Canada) according to the manufacturer’s instructions. RT-PCR–positive mosquito samples were used to validate our RNA shotgun metagenomic approach and to screen for the presence of additional arboviruses or co-infecting microorganisms. Detection of arboviruses (WNV, EEEV and SSHV) in mosquito pools was accomplished by real-time RT-PCR according to previously published methods [21–23]. To remove contaminating genomic DNA from the host and microbiota and thereby improve sensitivity for RNA virus detection, the extracted nucleic acids were pretreated with Turbo DNase (Thermo Fisher Scientific, Montreal, Canada). RNA was converted to DNA and uniformly amplified by multiple displacement amplification using the RepliG single cell WTA kit (Qiagen, Montreal, Canada). Equimolar amounts of DNA from each pool were used for library preparation using the Nextera XT DNA library preparation kit (Illumina, San Diego, CA, USA) following the manufacturer’s instructions. Sequencing was carried out on the MiSeq instrument (Illumina) using a MiSeq v3 600-cycle kit.

### Sequence analysis and metagenomic assessment

FASTQ sequence files were uploaded to the open-source, cloud-based metagenomic analysis platform CZ-ID (Chan Zuckerberg ID - Detect & Track Infectious Diseases) for global pathogen detection and surveillance [24]. Briefly, the CZ-ID metagenomic pipeline (v8.3) performed host read removal (*Culex pipiens*) and quality-filtering steps to eliminate nonspecific sequences, followed by assembly-based alignment and taxonomic classification using searches against the NCBI nucleotide (NT) and protein (NR) databases. Host filtering and quality control (QC) steps involved removal of sequencing adapters, short reads (< 35 bp), and low-quality reads (Q < 17) using a customized fastp workflow. Host-derived reads were identified and removed by aligning to the host reference genome using Bowtie2 and HISAT2, while duplicate reads were eliminated using CZID-dedup.

Taxonomic analysis and organism identification in CZ-ID were conducted using the sourmash compression-based approach [25], with sequence classification against the NCBI NT and NR databases. Taxonomic relative abundances were reported as reads per million (rPM) to facilitate the abundance comparison between taxa. A cutoff threshold of >25 rPM was applied to report taxonomic hits and exclude low-confidence detections.

For each sample processed through the CZ-ID platform, consensus genomes with the highest quality metrics were downloaded for further evaluation. This consisted in a two-stage quality-control process was employed: an initial screening to identify potentially publishable genomes among all assemblies (n = 71), followed by a secondary review to remove low-quality assemblies. Consensus sequences were assessed based sequencing depth (≥ 30x) and overall assembly quality (genome called ≥ 96%; at least 5 identical bases required for each position). Only consensus sequences meeting these criteria were retained for further analyses.

### Phylogenetic analysis and protein domain identification

Analyses were conducted using complete genome sequences or selected proteins inferred from predicted open reading frames (ORFs). To identify additional viral relatives for phylogenetic analyses, BLAST searches using viral sequences identified in this study was performed with NCBI databases. We also included when available, representative sequences described in the literature, particularly those with established genotype or taxonomic information, to better contextualize our findings. Putative ORFs were predicted from nucleotide sequences using the NCBI ORF Finder tool (https://www.ncbi.nlm.nih.gov/orffinder/), and the corresponding protein sequences were extracted for downstream analyses. Functional protein domain annotation was performed using InterProScan v106.0, available through the InterPro platform [26].

Consensus sequences for each group were aligned with MAFFT software v7.419 [27] using default values with flags –auto and --nuc/--amino depending on data type. Phylogenetic trees were constructed from all alignments using IQtree2 v2.2.0 [28] with options –seqtype DNA/AA depending on data type -m MFP (ModelFinder plus) with the GTR + I + G nucleotide substitution model and 100 bootstrap replicates. All trees were then annotated with metadata in iTOL website (iTOL: Interactive Tree Of Life) using the provided Excel template spreadsheet (iTOL_annotation_editor_v1_8_Excel.xlsm).

In total, 35 genomic sequences (including whole and segmented genomes) were deposited in the NCBI nucleotide database under the following accession numbers: PQ220305–PQ220316, PQ220318–PQ220326, PQ220329–PQ220331, and PX487090–PX487100.

## Results

We analysed the RNA metagenome of 16 mosquito pools comprising 348 adult female mosquitoes, including 263 *Culex pipiens*, 58 *Culiseta melanura* and 27 *Ochlerotatus stimulans* collected in the province of Québec between 2005–2016 (**Table 1**). Whole genome sequencing of mosquito homogenates generated 2.4 to 6 million total reads per sample. The raw reads were filtered through a series of CZID pipeline bioinformatic filters [24] yielding 0.4 to 2 million reads.

### Mosquito microbiota diversity

**Fig 2:**
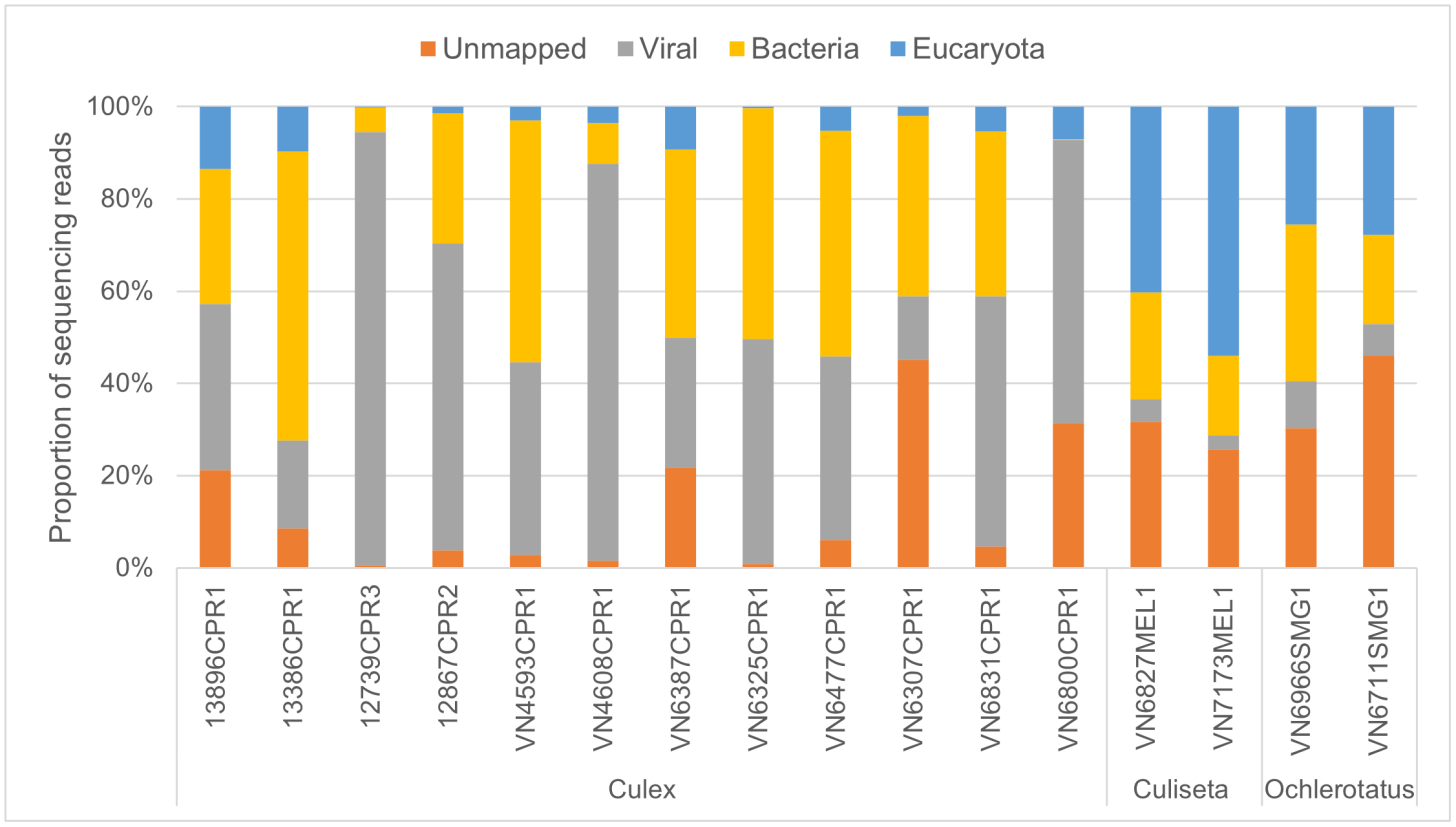
Taxonomic composition of sequencing reads across mosquito samples by genus.

The phylum diversity found in the mosquitoes was wide and included reads mapping to eukaryotes which were mostly fungi and parasites, bacteria and virus sequences found in the NCBI NT and NR databases (**Fig 2**). Unmapped reads to the current database were also noted with variable abundance within all mosquito species (1–46%). Prior to RNA extraction, samples were treated with DNase to remove genomic DNA, ensuring that sequencing libraries generated by reverse transcription captured RNA molecules exclusively. Consequently, bacterial and fungal sequences detected in this dataset represent ribosomal RNA (rRNA) and messenger RNA (mRNA) present in the RNA fraction, consistent with the transcriptional activity of known mosquito-associated microbiota, rather than genomic DNA [29,30]. The metagenome of *Culex pipiens* was particularly rich in viral reads comprising 14–94% of all sequences per sample compared to *Culiseta* and *Ochlerotatus* species which showed 3–10% viral content. Conversely, eucaryotic reads were more abundant in *Ochlerotatus* samples (26–28%) and highest in *Culiseta* samples which showed 40–54% of total reads. Bacterial sequence reads were on average similar amongst all mosquito species with 33% for *Culex*, 20% for *Culiseta* and 27% for *Ochlerotatus*.

**Fig 3:**
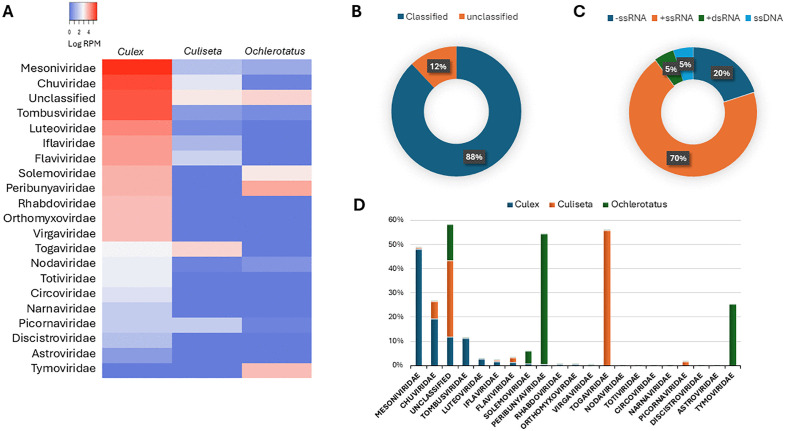
Virome diversity and relative abundance sorted by mosquito species. The figure shows the abundance of viral families expressed as a log of reads per millions **(A)**, the proportion of classified viral taxa **(B)**, genomic organization classification **(C)**, and viral families per mosquito species **(D)**.

Beyond the medically relevant arbovirus, we expected to find in our collected mosquitoes, we also detected sequence reads corresponding to viruses from several other families (**Fig 3A**). Taken together, the sequencing reads from all mosquito species collected in this study mapped to viral genomes classified in twenty viral families. Viruses of those families are hosted by a wide variety of organisms including protozoan, fungus, arthropods, vertebrates and plants. We also noticed that an important proportion (12%) of total reads from all mosquito species was mapping to viral species which were not thoroughly classified in the NCBI database (**Fig 3B**). Positive-sense single-strand RNA (ssRNA) was the most abundant class of detected viruses namely *Mesoviviridae*, *Tombusviridae*, *Luteoviridae*, *Iflaviridae*, *Flaviviridae*, *Solemoviridae*, *Tymoviridae*, *Virgaviridae*, *Togaviridae*, *Nodaviridae*, *Picornaviridae*, *Narnaviridae*, *Dicistroviridae* and *Astroviridae* (**Fig 3C**). We also identified negative sense ssRNA virus families with or without segmented genomes or circular genomes such as *Chuviridae*, *Peribunyaviridae*, *Rhabdoviridae* and *Orthomyxoviridae*. Finally, a double-strand RNA virus (*Totiviridae*) and a circular DNA virus (*Circoviridae*) were also detected.

The abundance of each virus taxon expressed as RPM per mosquito species and year of sample collection is presented in **Table 2**. In total, 60 viral taxa (using a threshold of ≥ 25 RPM) with identities to known viral species were detected using the *de novo* assembled viral contigs. Between 1 and 26 viral taxa were detected in *Culex* samples while the diversity was significantly lower in *Culiseta* and *Ochlerotatus* samples with five to nine viral hits. *Culex* samples with the highest number of viral species detected were collected in the cities of Laval and Montreal. Most detected viral species were unique, with some exceptions, notably the multiple species belonging to the *Mesoniviridae* and *Chuviridae* families. To investigate the presence of endogenous viral elements (EVEs), we performed nucleotide similarity searches against mosquito sequence databases and found no evidence of their existence.

**Table 2:** Detection and abundance of viral taxa in three mosquito species from 2005 to 2016 expressed as reads per million (RPM).

		*Culex pipiens*												*Culiseta melanura*		*Ochlerotatus stimulans*		
Taxonomy	Species	12739CPR3	12867CPR2	13386CPR1	13896CPR1	VN4608CPR1	VN4593CPR1	VN6387CPR1	VN6477CPR1	VN6325CPR1	VN6307CPR1	VN6831CPR1	VN6800CPR1	VN7173MEL1	VN6827MEL1	VN6711SMG1	VN6966SMG1	No detection
		2005	2005	2005	2005	2014	2014	2015	2015	2015	2015	2016	2016	2016	2016	2016	2016	
Bunyavirales*	Culex Bunyavirus 2	0	0	0	0	58	51	0	0	0	0	338	43	0	0	0	0	4
Bunyavirales*	Culex Bunya-like virus	0	0	0	0	290	115	0	0	0	0	497	309	0	0	0	0	4
Chuviridae	Imjin mivirus	0	0	0	0	0	0	0	0	0	0	257	0	0	45	0	0	2
Chuviridae	Wuhan mosquito virus 8	0	0	0	0	0	0	0	0	0	0	198	0	0	40	0	0	2
Chuviridae	Culex mosquito virus 5	12793	17019	0	0	433	0	76	0	0	0	0	0	0	47	0	0	5
Chuviridae	**Culex mosquito virus 4**	73481	116415	0	111	4325	0	222	0	0	0	574	0	34	179	0	0	8
Circoviridae	Mosquito associated circovirus 1	0	0	0	0	0	0	0	169	0	0	0	0	0	0	0	0	1
Flaviviridae	**West Nile virus**	27	0	3432	0	0	536	136	1122	160	1055	443	0	0	0	0	0	8
Flaviviridae	**Culex flavivirus**	0	0	0	0	179	253	526	4528	167	512	2354	50	0	0	0	0	8
Flaviviridae*	Culiseta flavivirus	0	0	0	0	0	0	0	0	0	0	0	0	75	0	0	0	1
Iflaviridae	Sacbrood virus	0	0	0	0	0	0	41	0	0	0	0	0	0	0	0	0	1
Iflaviridae*	Yongsan iflavirus 1	0	0	0	0	0	0	0	75	0	0	0	0	0	0	0	0	1
Iflaviridae*	Aedes Ifla-like virus	0	0	0	0	369	0	0	0	0	0	0	0	0	0	0	0	1
Iflaviridae*	**Culex Iflavi-like virus 4**	0	0	0	0	19404	0	0	0	0	0	0	0	0	0	0	0	1
Marafivirus*	Sorghum bicolor marafivirus	0	0	0	0	0	0	0	0	0	0	0	0	0	0	94	0	1
Marafivirus*	Grapevine rupestris vein feathering virus	0	0	0	0	0	0	0	0	0	0	0	0	0	0	0	450	1
Mesoniviridae	Odorna virus	930	0	0	0	832	0	0	0	0	0	0	0	0	0	0	0	2
Mesoniviridae	**Alphamesonivirus 1**	339775	0	0	0	125497	0	0	0	0	0	86612	0	28	0	0	0	4
Mesoniviridae	Alphamesonivirus 3	214	0	0	0	48	0	0	0	0	0	0	0	0	0	0	0	2
Mesoniviridae	Alphamesonivirus 10	1715	0	0	0	816	0	0	0	0	0	77	0	0	0	0	0	3
Mesoniviridae	Alphamesonivirus 2	146	0	0	0	74	0	0	0	0	0	0	0	0	0	0	0	2
Mesoniviridae	Alphamesonivirus 4	153	0	0	0	0	0	0	0	0	0	0	0	0	0	0	0	1
Mesoniviridae	Alphamesonivirus 5	303	0	0	0	187	0	0	0	0	0	67	0	0	0	0	0	3
Negevirus	**Negev virus**	7023	0	0	0	0	0	0	0	0	0	0	0	0	0	0	0	1
Negevirus	Culex Biggie-like virus	81	0	0	0	26	31	0	0	0	0	0	0	0	0	0	0	3
Negevirus	**Biggievirus Mos11**	5155	0	0	0	1899	1993	0	0	0	0	0	0	0	0	0	0	3
Negevirus	Biggie virus	59	0	0	0	0	0	0	0	0	0	0	0	0	0	0	0	1
Nodaviridae*	Culex Negev EO-329-like virus	83	0	0	0	36	0	0	0	0	0	0	0	0	0	0	0	2
Nodaviridae*	Culex Negev #730-like virus	135	0	0	0	0	0	0	0	0	0	0	0	0	0	0	0	1
Nodaviridae*	Culex Virga-like virus	1617	876	0	0	0	0	0	0	0	0	0	0	0	0	0	0	2
Nodaviridae*	**Culex mosquito virus 1**	19825	0	0	454	45376	4768	1401	3478	20382	0	0	0	0	0	0	0	7
Orthomyxoviridae	**Wuhan Mosquito Virus 6**	0	0	0	0	1464	903	47	0	0	0	3365	537	0	0	0	0	5
Peribunyaviridae	Culex pseudovishnui bunya-like virus	0	0	0	0	0	0	0	0	0	0	31	0	0	0	0	0	1
Peribunyaviridae	Bunyaviridae environmental sample	0	0	0	0	894	270	32	0	0	0	4818	780	0	0	0	0	5
Peribynyaviridae	**Snowshoe hare virus**	0	0	0	0	0	0	0	0	0	0	0	0	0	0	11153	0	1
Peribynyaviridae	California encephalitis orthobunyavirus	0	0	0	0	0	0	0	0	0	0	0	0	0	0	57	0	1
Peribynyaviridae	La Crosse orthobunyavirus	0	0	0	0	0	0	0	0	0	0	0	0	0	0	28	0	1
Picornavirales*	Culex picorna-like virus 1	0	0	0	0	1132	0	0	0	0	0	0	0	0	0	0	0	1
Picornaviridae*	Jotan virus	0	0	0	0	0	0	0	67	0	0	0	0	0	0	0	0	1
Picornaviridae*	Yongsan picorna-like virus 1	36	0	0	0	0	0	0	0	0	0	0	0	0	0	0	0	1
Picornaviridae*	Ista virus	0	0	0	0	0	0	0	0	0	0	0	0	46	31	0	0	2
Rhabdoviridae	**Flanders hapavirus**	0	0	0	0	1040	0	0	0	0	0	5651	0	0	0	0	0	2
Solemoviridae*	Atrato Sobemo-like virus 1	0	0	0	0	0	0	0	0	0	0	0	0	0	0	474	663	2
Solemoviridae*	Guangzhou sobemo-like virus	1439	1441	0	0	2978	1358	180	0	0	0	1485	614	0	0	0	0	7
Togaviridae	**Eastern equine encephalitis virus**	0	0	0	0	0	0	0	0	0	0	0	0	1393	1189	0	0	2
Tolivirales*	**Marma virus**	2068	5293	0	29	7744	2925	4447	0	0	0	8094	2290	0	0	0	0	8
Tombusviridae*	**Culex associated Tombus-like virus**	40556	0	0	511	68037	5886	2669	1979	11376	0	73	0	0	0	0	0	8
Totiviridae*	Totivirus-like Culex mosquito virus 1	95	310	0	0	0	0	0	0	0	0	0	0	0	0	0	0	2
Tymovirales*	Bat tymo-like virus	0	0	0	0	0	0	0	0	0	0	0	0	0	0	234	71	2
Tymoviridae	Grapevine fleck virus	0	0	0	0	0	0	0	0	0	0	0	0	0	0	0	648	1
Tymoviridae	Chayote mosaic virus	0	0	0	0	0	0	0	0	0	0	0	0	0	0	1026	1580	2
Tymoviridae	Watercress white vein virus	0	0	0	0	0	0	0	0	0	0	0	0	0	0	184	78	2
Tymoviridae*	Bee Macula-like virus	0	0	0	0	0	0	0	0	0	0	0	0	0	0	140	0	1
Tymoviridae*	Fig fleck-associated virus 2	0	0	0	0	0	0	0	0	0	0	0	0	0	0	0	1039	1
Unclassified	**Culex negev-like virus 1**	634	280	0	25	0	0	0	0	0	0	0	0	0	0	0	0	3
Unclassified	Hubei mosquito virus 4	0	0	0	0	0	0	0	0	0	1531	0	0	0	0	0	0	1
Unclassified	Hubei virga-like virus 2	0	0	0	0	0	0	0	0	0	0	334	0	0	0	0	0	1
Unclassified	Culex inatomii luteo-like virus	59	371	0	0	1445	76	0	0	0	0	383	56	0	0	0	0	6
Unclassified	Hubei mosquito virus 2	240	707	0	0	1230	460	26	0	0	0	1533	163	0	0	0	0	7
Virgaviridae*	Atrato Virga-like virus 2	0	0	0	0	0	0	0	0	0	0	2212	0	0	0	0	0	1
Wolbachia		21846	52381	10561	699	22318	20961	6110	10942	29641	6242	70295	0	0	0	0	0	11
Spiroplasma		0	0	0	0	0	0	0	0	0	0	0	0	0	0	45332	15745	2

* Taxa not thoroughly classified in the genus, family or order in the NCBI database. Taxa with complete genomes are depicted in bold. Bacteria reads are shown in bold brackets.

We observed many viral species which are known to specifically infect plants and some of which are transiently found in insects (carrier) feeding on plants [31]. Plant viruses are classified in *Tymoviridae*, *Virgaviridae*, *Solemoviridae*, *Luteoviridae* or *Tombusviridae* families. Interestingly, *Ochlerotatus stimulans* exhibited 5 and 26% of totals reads for plant viruses classified in *Solemoviridae* and *Tymoviridae*, respectively (**Fig 3D**). Some viral species of the *Solemoviridae* are transmissible by insects such as leafhoppers or beetle or can be transmitted mechanically between plants. However, mosquitoes were not reported to be hosts of these viruses [32]. Although at low levels, we detected mycoviruses (viruses of fungi) which are classified in *Totiviridae* and *Narnaviridae* families in *Culex pipiens*.

We detected a total of 82 bacterial genera in all mosquito species (S1 Table). The most prevalent genus was *Wolbachia* followed by *Spiroplasma*. We detected *Wolbachia* reads in all but one of the *Culex* samples, and none in *Culiseta* or *Ochlerotatus* samples. *Wolbachia* are intracellular, Gram-negative endosymbiotic bacteria found in many arthropods and filarial nematodes [33]. Depending on the host species and strain, they can establish either parasitic or mutualistic associations. *Spiroplasma* reads were only found in *Ochlerotatus* mosquitoes. *Spiroplasma sp.* are helically shaped, motile and wall-free bacteria thriving as endosymbiont of insects modulating reproductive mechanism of the host [34] or conferring protection against parasitic organisms [35]. A few *Spiroplasma sp.* are pathogenic to agriculturally important plants such as corn causing the corn stunt disease while other species are pathogenic to honeybees [36]. Human infections involving *Spiroplasma sp.* are scarce. However cases of uveitis in children [37] and systemic infection in a immunodeficient patient [38] were reported.

We also detected a wide array of eukaryotic organisms in our mosquito samples (S2 Table). Several species of *Microsporidia* were present, some of which are known to be mosquito parasites. For example, members of the genera *Amblyospora* and *Vavraia* are intracellular *Microsporidia* that infect mosquito larvae following ingestion of infectious spores. Biocontrol strategies of mosquito populations using *Microsporidia* were also studied but only a few field studies have been conducted with polymorphic *Microsporidia* [39].

The sequencing metrics for the 35 complete or near complete genomes are presented in S3 Table.

### Detection and Phylogeny of Arboviruses

Eight WNV RT-PCR positive *Culex pipiens* samples out of eleven (72%) were detected (27–3,432 RPM) by the RNA metagenomic sequencing method (**Table 3**). However, only four WNV genomes were entirely resolved (samples VN6477 and VN6307) or near completion (samples 13386 and VN4593). The percentage of WNV genome assembled varied from 6 to 100% compared to the best WNV reference genome matched sequences. Three *Culex* samples did not generate any significant amount of WNV sequence reads. Two of these samples (12867 and VN4608) had high Ct values (≥ 31) while one sample (13896) had a low Ct value (**Table 3**). The absence of detectable reads for samples 12867 and 4608 is most likely attributable to low viral loads, whereas partial RNA degradation is a more plausible explanation for sample 13896, which exhibit lower RPM values across several taxa compared to other *Culex* samples (Table 2). The WNV negative RT-PCR *Culex* sample did not generate any specific reads for WNV thus validating the specificity of the RNA metagenomic sequencing method.

**Table 3:** Real time RT-PCR detection of arboviruses in mosquito pools and metagenomic sequencing using extracted nucleic acid.

Samples	Arbovirus surveillance screening			RNA Metagenomic sequencing			
	Virus detected	target gene	RT-PCR (Ct)	rPM	% Genome called	Coverage dept (fold)	Sequence lenght (nt)
13896CPR1	WNV	*Env*	15.9	0	NA	NA	NA
13386CPR1			16.4	3432	100	113	10 373
12739CPR3			20.6	27	6	1	NA
12867CPR2			35.7	0	NA	NA	NA
VN4593CPR1			21.3	536	96	31	10 404
VN4608CPR1			30.9	0	NA	NA	NA
VN6387CPR1			19.8	79	69	12	NA
VN6325CPR1			21,0	160	84	14	NA
VN6477CPR1			21.2	439	100	90	10 781
VN6307CPR1			23.9	297	99	91	10 696
VN6831CPR1			23.4	443	88	21	NA
VN6800CPR1	WNV neg		ND	0	NA	NA	NA
VN6827MEL1	EEEV	*E3*	13.1	1189	100	84	11 604
VN7173MEL1			31,0	1393	100	134	11 611
VN6966SMG1	SSHV*	*N*	15.4	0 (S)	NA	NA	NA
VN6711SMG1			17.4	78 (S)	100	97	578
VN6711SMG1		NT	NT	5772 (M)	100	1058	4 428
VN6711SMG1			NT	2782 (L)	100	317	6 882

NA, not available; NT, not tested; ND, not detected; Ct, cycle threshold; nt. nucleotide; WNV, West Nile virus; EEEV, Equine encephalitis virus; SSHV, Snowshoe hare virus; L, large segment; M, medium segment; S, small segment; rPM; reads per million. % genome called measures how much of the genome is covered (breadth); Coverage folds measures how deeply the genome is covered (depth).

**Fig 4:**
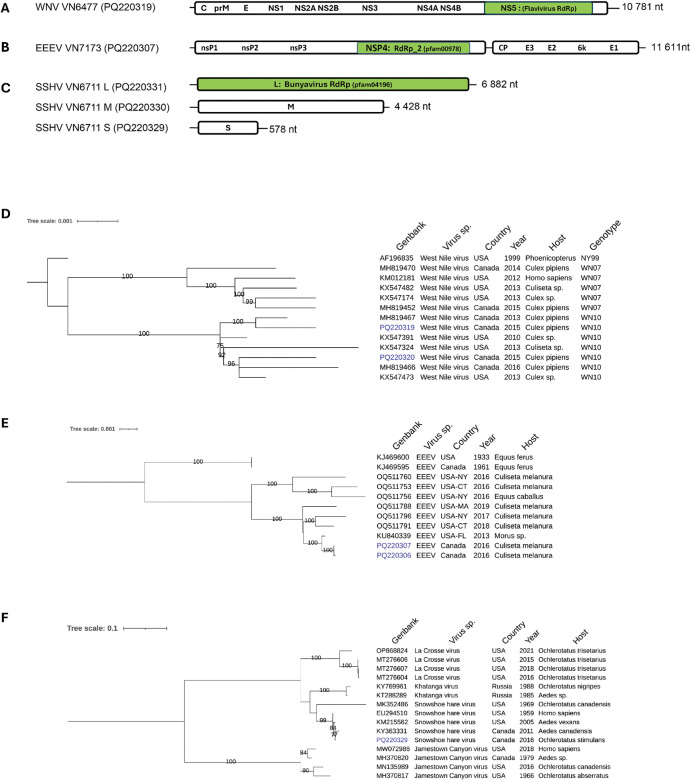
Genomic organization and maximum likelihood, midpoint-rooted phylogenetic trees of arboviruses. Genomic structure and phylogenetic trees of West Nile virus **(A, D)**, Eastern equine encephalitis virus (B, E) and Snowshoe hare virus (C, F) using whole genomes. Virus identified in this study are highlighted in blue. The scale bars indicate the number of nucleotide substitutions per site. C, capsid protein; prM, premembrane protein; E, envelope protein; NS, non-structural protein; RdRp, RNA-dependent RNA polymerase; L, large segment; M, medium segment; S, small segment.

The genomic structure of WNV consists of a monopartite positive-sense single-stranded RNA (+ssRNA) of ∼ 11 kb coding for a single large polyprotein (**Fig 4A**). Sample 13386 was 100% identical to a WNV partial sequence collected in 2005 in Canada (MH819451). Both samples 13386 and VN4593 had partial genomic sequences and were excluded from the phylogenetic analysis. Two near complete WNV genome (99%) were assembled (PQ220319 and PQ220320) and used for a phylogenetic assessment of their relationship to other publicly available WNV genomes from North America. The two WNV genomes isolated in the province of Québec clustered and formed a distinct clade with WNV sequences of the WN10 genotype (**Fig 4D**). Among those, two sequences MH819466 and MK819467 were previously reported in a longitudinal study of WNV evolution in Canada [40]. Both Québec isolates share 99,2% nucleotide identity to the firstly introduced WNV strain in North America (NY99).

Positive EEEV *Culiseta* samples, both collected in 2016, obtained a high number of reads (∼1000 RPM) (**Table 3**). The genomic structure of EEEV is characterized by a monopartite +ssRNA of ∼ 12 kb with two open reading frames (ORF). These ORFs are coding for non-structural and structural polyproteins (**Fig 4B**). Two EEEV genomes, with 99,8–99,9% completeness (PQ220307 and PQ220306), were assembled and analysed alongside a set of closely related genome sequences from the USA and Canada for phylogenetic analysis. The two Québec strains formed a distinct clade with a 2013 WNV American strain isolated from a sea bird (*Morus* sp) in Florida (**Fig 4E**). Québec strains are 99,9% identical at the nucleotide level (1 nucleotide substitution in the *E1* gene) and they diverge from the 2013 Florida isolate by a subtle difference of 11 nucleotide substitutions.

The SSHV genome is constituted of a tripartite negative ssRNA, each segment coding for a different protein namely the capsid protein, membrane glycoprotein and RdRp (**Fig 4C**). Among the two SSHV RT-PCR positive samples, one sample (VN6711) showed varying levels of sequencing reads (78–5772 rPM) for the S, M and L segments (**Table 3**). Complete genome resolution of the three segments was achieved with 97-to-1058-fold coverage depth. The entire nucleotide sequence of the S fragment (PQ220329) was used for phylogenetic analysis using available SSHV sequences and sequences from related taxon such as La Crosse virus and Jamestown canyon virus which are members of the *Orthobunyavirus* genus. All SSHV clustered in a single clade including isolates collected from 1959 to 2016 (**Fig 4F**). Interestingly, the Québec sample had 98,96% nucleotide identity with a strain isolated from a Snowshoe hare (*Lepus americanus*) in 1959 in Montana, USA. Only six nucleotide substitutions difference was noted between the two isolates indicating a very slow acting evolutionary process in North America. Conversely, another American strain of SSHV isolated from *Ochlerotatus canadensis* collected in 1969 (MK352486) in the Hockomock swamp located in the state of Massachusetts showed less nucleotide identity (85,42%) with the Québec strain arguing in favour of the existence of viral variants in different geographical locations. The Québec strain also shared high nucleotide identity (98,6%) with another Canadian isolate collected in 2011 (KY363331) from *Aedes vexans,* a bridge vector for WNV. The Québec SSHV taxon is distantly related to Khatanga virus (∼87%) found in Russia and La Crosse viruses (∼83%) located in the United States. The Jamestown canyon virus is more distantly related to SSHV forming a distinct clade with nucleotide identities varying around 75–77% with SSHV.

We did not detect other known arboviruses besides WNV, EEEV and SSHV which were predicted to be found in our mosquito samples.

### Viral metagenome assessment

Some viral species detected in this study from mosquito microbiota are classified in viral families with known vertebrate pathogens and others are known to be found specifically in insects or plants. It is not known at this point if the viruses detected in this study are hosted by other organisms found in the province of Québec as part of an enzootic/sylvatic cycle. The following sections focus on viruses for which we obtained complete or near-complete genomes.

#### Negative-sense, single-stranded RNA viruses.

**Fig 5:**
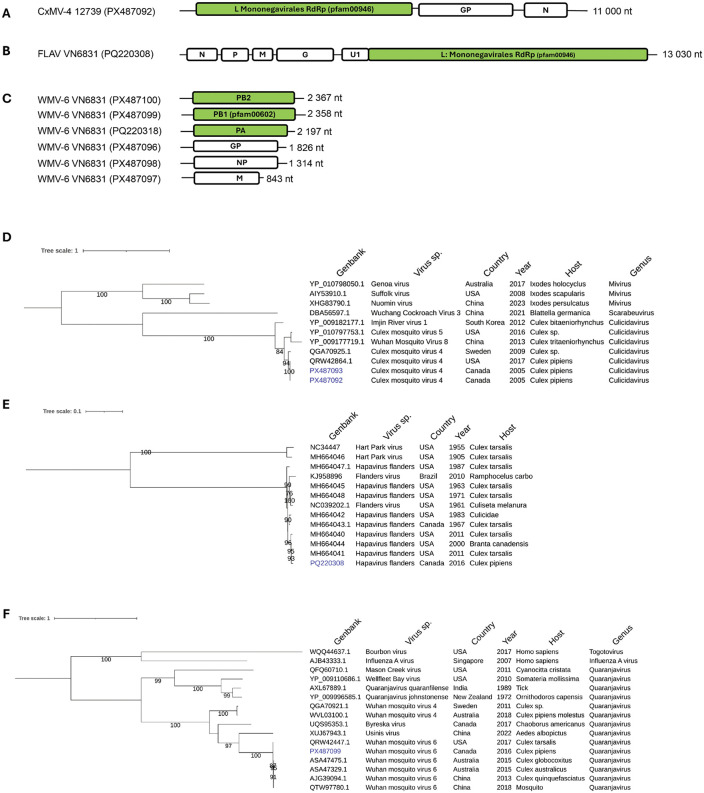
Genomic organization and maximum likelihood, midpoint-rooted phylogenetic trees of negative single strand RNA viruses. Genomic structure and phylogenetic trees of Culex mosquito virus 4 (A, D) and Flanders virus (B, E) using whole genome and Wuhan mosquito virus 6 using the PB1 protein sequence inferred from segment 2 **(C, F)**. Virus identified in this study are highlighted in blue. The scale bars indicate the number of nucleotides or amino acid substitutions per site. L, Large polymerase protein; G and Gp, glycoprotein; N, nucleocapsid; P, phosphoprotein; M, matrix protein; U1, accessory protein; PB1 and PB2, polymerase basic protein; PA, polymerase acidic protein; NP, nucleoprotein.

**Chuvirus:** Viral species classified in the *Chuviridae* family constituted 20% of total viral reads in *Culex pipiens* and 7% in *Culiseta melanura* (**Fig 3D**). No significant number reads were found in *Ochlerotatus stimulans*. The *Chuviridae* family includes 16 genera and over 40 species capable of infecting several terrestrial or marine arthropods and vertebrate species such as fish, reptiles and mammals [41,42]. The genomic structures of viral members of the *Chuviridae* are diverse and include segmented, non-segmented and circular -ssRNA of 6–11 kb with three ORFs. The taxon Culex mosquito virus 4 (CxMV-4) of the *Culicidavirus* genus was detected in six *Culex* and two *Culiseta* samples with varying levels of reads ranging from 34 to 116 415 RPM (**Table 2**). Two complete monopartite CxMV-4 genomes of 11 kb (PX487092 and PX487093) were assembled (**Fig 5A**). Blast analysis of the Québec sequences revealed high nucleotide identity (99,8%) with a CxMV-4 sequence (MH188031.1) derived from a *Culex* sample collected in 2016 in California, USA. Phylogenetic analysis of the deduced RdRp protein was performed with representatives of three *Chuviridae* genera namely *Culicidavirus*, *Scarabeuvirus* and *Mivirus*. A robust clade was observed between the Québec strains and isolates from collected in the USA and Sweden in 2017 and 2009 respectively (**Fig 5D**). Only two amino acids difference in the RdRp protein was observed between the Québec strains and the 2017 American isolate (QRW42864). Culex mosquito virus 5, Wuhan mosquito virus 8 and Imjin river virus showed less amino acid identities (80–89%) to the Québec strains despite being members of the *Culicidavirus* genus. Viral members of the two other genera *Scarabeuvirus* and *Mivirus* were significantly divergent with the Québec strains with 33–36% amino acid identities.

**Hapavirus:** Flanders virus (FLAV) is a mosquito-borne virus classified in the genus *Hapavirus* (family *Rhabdoviridae*). Similarly to WNV, FLAV is maintained in nature through a mosquito-avian cycle involving *Culex* and *Culiseta* mosquito species and passerine birds as reservoirs [43]. We detected reads mapping to FLAV sequence in two *Culex* samples (4608 and 6831) (**Table 2**). The closest relative with the Québec strain was a FLAV sequence (MH664044) isolated from a Canada goose (*Branta canadensis*) in the USA sharing 99,3% nucleotide identity. A complete FLAV genome (**Fig 5B**) was fully resolved for sample VN6831 (PQ220308) and used along a set of North American FLAV strains and two Hart Park virus sequences for phylogenetic analysis (**Fig 5E**). All the FLAV sequences formed a well-resolved clade (**Fig 5E**). These sequences are derived from viral isolates from mosquitoes or birds collected between 1967 and 2016 and showing 96,7–99,3% nucleotide identities with the Québec strain. The two Hart Park viruses formed an outgroup clade showing 77% nucleotide identities.

**Wuhan mosquito virus 6:** Wuhan mosquito virus 6 (WMV-6) is classified in the genus *Quaranjavirus*, family *Orthomyxoviridae*. WMV-6 genetic signature was detected (47–3365 RPM) in five *Culex* samples collected in 2014 and beyond (**Table 2**). This taxon was not detected in *Culiseta* and *Ochlerotatus* mosquitoes. The *Orthomyxoviridae* is a large family including pathogenic and non-pathogenic viruses classified in six genera. *Quaranjavirus* includes virus hosted by various arthropods and vertebrate hosts. The genome of WMV-6 is composed of six segments of -ssRNA (**Fig 5C**). The three longest segments are coding for the replication protein complex formed by PB2, PB1 and PA proteins while the other segments codes for structural virion proteins.

Five of the six genomic segments of the WMV-6 Québec strain shared high nucleotide identities (97,3–98,4%) with Australian sequences from *Culex* collected in 2015 (MF176253.1, MF176336.1, MF176381.1, MF176378.1, MF176252.1) while the PB1 segment (*RdRp* gene) has a nucleotide identity of 98,1% with a Chinese sequence (KM817625.1) isolated in 2013 from *Culex quinquefasciatus.* PB1 protein sequence was inferred from the ORF (*RdRp* gene) of segment 2 of the Québec strain VN6831 (PX487099) and used for phylogenetic assessment using several WMV-6 strains along with eight other *Quaranjavirus* and two representatives of the genera *Influenza virus A* and *Thogotovirus*, respectively.

A well-resolved clade was formed by all WMV-6 strains collected in China, Australia, the USA and Canada between 2013 and 2016 (**Fig 5F**). The PB1 sequences varied from 98,5–99% amino acid identities and had only a few amino acid substitutions (8–12) despite being collected in different continents. Furthermore, all the WMV-6 strains reported in the NCBI database were isolated from mosquito hosts. The Usinis virus was the closest relative to WMV-6 sharing 60% identity followed with the Byreska virus (54%) and two Wuhan mosquito viruses 4 (53%). The third group of *Quanjavirus* genomes formed several subclades and their PB1 sequences diverged significantly (33−35%) from the WMV-6 taxa. Finally, the human pathogen Influenza A virus and Bourbon virus, two members of the *Orthomyxoviridae* had highly divergent PB1 sequences with WMV-6.

#### Positive-sense, single-stranded RNA viruses.

**Fig 6:**
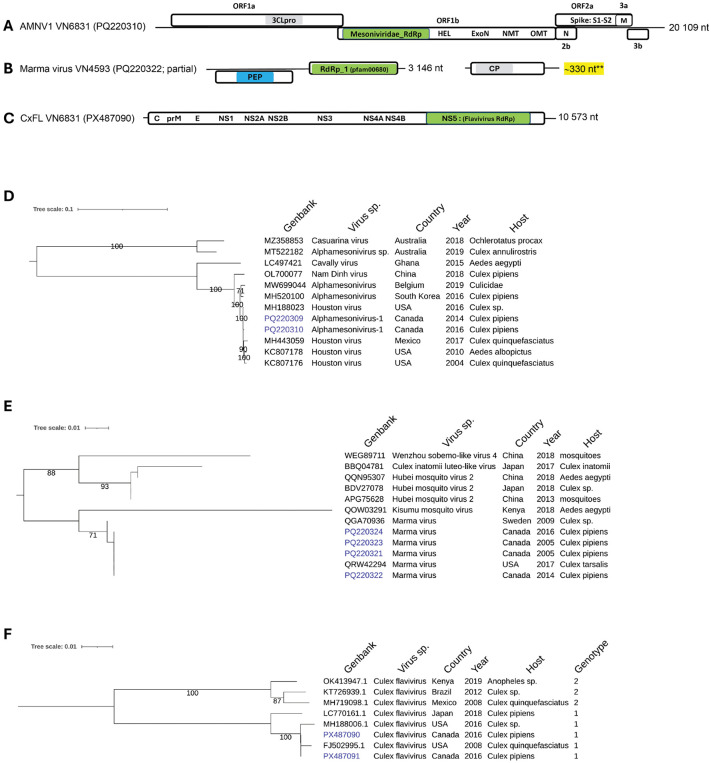
Genomic organization and maximum likelihood, midpoint-rooted phylogenetic trees of positive single strand RNA viruses. Genomic structure of AMNV-1 **(A)** Marma virus (B) and CxFL. Phylogeny using whole genome of AMNV-1 **(D)** CxFL (F) and RdRp of Negev virus **(E)**. Virus identified in this study are highlighted in blue. 3CLpro, 3C-like protease; RdRp, RNA-dependent RNA polymerase; HEL, helicase; ExoN, exonuclease; NMT and OMT; methyltransferases; N, nucleoprotein; M, matrix protein; PEP, peptidase; CP and C, capsid protein; prM, premembrane protein; E, envelope protein, NS, non-structural proteins. The scale bars indicate the number of nucleotides or amino acid substitutions per site.

**Mesonivirus:** The most prevalent and abundant viral species detected in *Culex pipiens* are members of the *Mesoniviridae* family with 48% of total viral reads and 500,000 RPM (**Fig 3A and 3D**). This proportion was significantly lower (below 1%) for *Culiseta melanura* and *Ochlerotatus stimulans*. We detected over 100,000 RPM attributed to the taxon Alphamesonivirus-1 (AMNV-1) in three *Culex* samples collected in 2005, 2014 and 2016 from traps located in Montréal and Laval cities (**Table 2**). One *Culiseta* sample showed a modest 28 RPM for this taxon. *Alphamesonivirus* are classified in the *Mesoniviridae* family of the *Nidovirales* order. These viruses are known to specifically infect insects worldwide and are therefore considered as ISVs. The genomic structure of Alphamesonivirus is characterized by a monopartite +ssRNA of ∼ 20 kb with seven ORFs [44]. The first two ORFs are coding for non-structural proteins while the structural proteins are coded by sub genomic RNA (**Fig 6A**). The two Québec sequences PQ220309 (2014) and PQ220310 (2016) were 99,78% identical at the nucleotide level with 43 nucleotides substitutions. Phylogenetic analysis of these two sequences with Mesonivirus sequences collected worldwide revealed a well resolve clade with Houston virus isolates from the United States and Mexico (**Fig 6D**). The nucleotide identity between these sequences was very high ranging from 99,4–99,8%. The Québec lineage diverge significantly with the Cavally sequence (91,4%) from Ghana collected in 2015 and the Australian sequences (80%) isolated in 2018–2019.

**Marma virus:** We detected specific reads to the Marma virus taxon in eight *Culex* samples and none in *Culiseta* or Ochlerotatus (**Table 2**). The Marma virus (unclassified *Tolivirale*) was detected for the first time in *Culex* in Sweden in 2009 and in the United States afterwards in *Culex tarsalis* mosquitoes. Recently, other hosts such as the tick *Otobius megnini* [45] and the leafhopper *Empoasca fabae* [46] were reported to harbour Marma viruses enlarging the host spectrum to other insects.

We used an assembled contig of ∼ 3146 nt from VN4593 sample for Blast analysis. The highest score sequence was obtained with a Marma virus sequence from a *Culex* mosquito collected in Sweden (MK440646) showing 98,3% nucleotide identity. The Marma virus genome is bi segmented. We entirely resolved the first segment and only 20% of the second segment. The first segment consists of two ORFs, the first coding for a trypsin-like protease (SSF50494) followed by the ORF for RdRp (pfam00680) (**Fig 6B**). The second segment which has only one ORF coding for the capsid protein. A phylogenetic tree was constructed using available RdRp sequences inferred from Marma virus genomes and other related viruses found by Blastp analysis. All North American RdRp sequences formed a distinct clade and were 100% conserved while only one amino acid difference was noted with the 2009 Swedish isolate (**Fig 6E**). Another well-resolved clade was formed between Wuhan mosquito virus 2 (WMV2) strains including the Culex inatomii luteo-like virus, all showing ∼93% amino acid identities with the Canadian strains.

**Culex flavivirus:** Another viral member of *Flaviviridae* was detected in *Culex* mosquitoes namely Culex flavivirus (CxFV), an insect-specific virus unable to replicate in vertebrate cells as opposed to the West Nile virus, a flavivirus (arbovirus) which can replicate in vertebrate and arthropod cells. Six out of twelve *Culex* samples showed the presence of both WNV and CxFV based on detected specific reads, while two samples were positive only for CxFL. (**Table 2**). The closest relative to the Québec CxFV strains was a sequence (MH188006) from *Culex* collected in the USA in 2016 with 99,62% nucleotide identity. Two near-complete CxFL genomes (PX487090 and PX487091) were used along a set of CxFV genomic sequences to build a phylogenetic tree. The Québec sequences clustered in a clade which includes CxFV genotype 1 sequences from the United States and Japan sharing between 98 and 99% nucleotide identities (**Fig 6F**). The other CxFV distinct clade was formed by genotype-2 sequences from Kenya, Brazil and Mexico, all sharing ∼ 90% nucleotide identities with the Québec strains.

**Tombus-like virus:** Members of the Tombusviridae were the third most prevalent viral taxa found in *Culex pipiens* (11% of total reads) with over 100 000 RPM (**Fig 3A and 3D**). *Culiseta* and *Ochlerotatus* species did not show any significant number of reads. The *Tombusviridae* family includes mostly monopartite viruses of 3.7 to 4.8 kb which typically infect plants [47].

**Fig 7:**
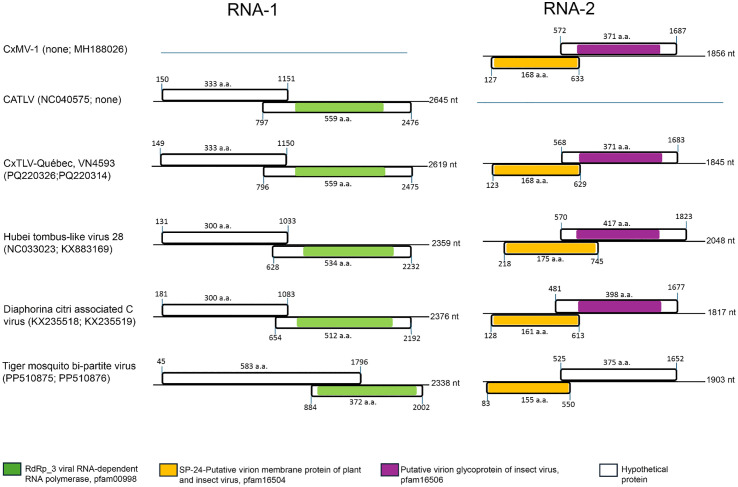
Genome organization and protein domain annotation of Tombus-like viruses. Comparison of the genomic organization between Culex mosquito virus 1, Culex associated tombus-like virus and Culex tombus-like virus, strain Québec as well as three bi-segmented viruses. The bipartite genome of Hubei tombus-like virus 28, Diaphorina citri associated C virus and Tiger mosquito bipartite virus are also depicted in the figure to illustrate the genes organization and the presence of similar protein domains. All of these viruses are not currently classified within ICTV but share some relatedness with Tombus-related insect viruses. The first and last nucleotide number are indicated above the ORF along with the number of amino acids for encoded proteins. The length in nucleotides of each genomic segment is indicated at the end. Accession numbers of both RNA-1 and RNA-2 are presented in parentheses.

**Fig 8:**
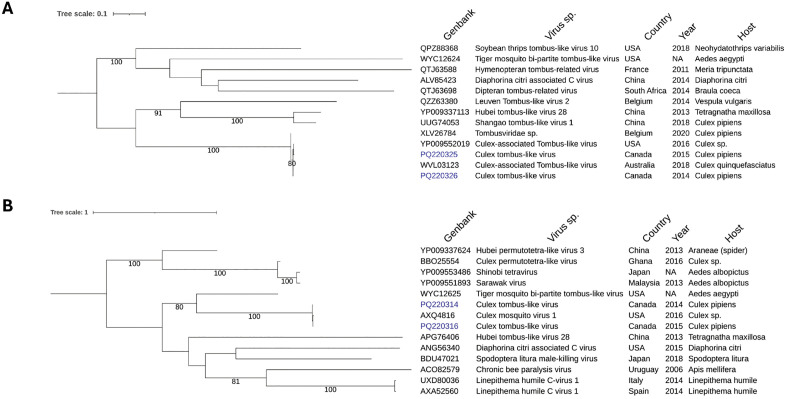
Maximum likelihood, midpoint-rooted phylogenetic trees of Culex Tombus-like viruses. The figure shows the phylogeny of RdRp (A) and SP- 24 (B) proteins inferred from Culex tombus-like viruses. Virus identified in this study are highlighted in blue. The scale bars indicate the number of amino acid substitutions per site.

*Culex*-associated tombus-like virus (CATLV) was detected in eight *Culex* pipiens samples collected between 2005 and 2016 (**Table 2**). CATLV is currently not thoroughly classified within the *Tombusviridae* and differs significantly from the canonical genome organization and the type of protein found in *Tombusvirus*. A 2,619 nt contig assembled from sample VN4593 showed 99.1% nucleotide identity with a CATLV strain from the USA (NC040575.1). The contig contains two overlapping ORFs encoding a hypothetical protein and an RNA-dependent RNA polymerase (RdRp; pfam00998) (**Fig 7**). RdRp sequences from Québec isolates clustered in a well-supported CATLV clade and shared 99.3–99.8% amino acid identity with other CATLV strains, but only 35.6–50% amino acid identity with more distantly related tombus-like insect viruses (**Fig 8A**).

In parallel, ~ 1.8-kb contigs encoding a virion membrane protein (SP-24; pfam16504) and a glycoprotein (pfam16506) were detected in seven Québec samples (**Table 2**) and matched Culex mosquito virus 1 (CxMV-1; MH188026) (Fig 7). SP-24 sequences from Québec strains clustered with the only CxMV-1 (98.8% amino acid identity) found in the NCBI database and were clearly distinct from SP-24 of Hubei tombus-like virus 28 (~40% identity) (**Fig 8B**).

Although CATLV and CxMV-1 were originally described as separate viruses [48], their consistent co-detection in the same samples, comparable read abundances (**Table 2**), and very high protein sequence identity (98.8–99.9%) with proteins encoded by the Québec sequences raise the possibility that they represent genomic segments of a single bi-segmented virus. However, the alternative hypothesis of two distinct but co-circulating viruses cannot be excluded. Pending experimental confirmation, we provisionally designate this putative virus Culex tombus-like virus (CxTLV), strain Québec.

**Fig 9:**
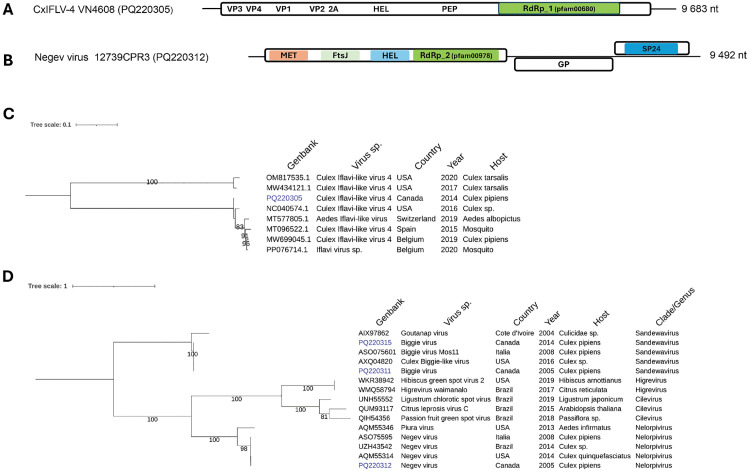
Genomic organization and maximum likelihood, midpoint-rooted phylogenetic trees of positive single strand RNA viruses. Genomic structure of Culex Iflavi-like virus-4 (A) and Negev virus **(B)**. Phylogeny using whole genome of Culex Iflavi-like virus (C) and RdRp of Negev virus **(D)**. Virus identified in this study are highlighted in blue. VP, viral proteins; HEL, helicase; PEP, peptidase; RdRp, RNA-dependent RNA polymerase; MET, methylase; FTsJ, membrane-associated protein; GP, glycoprotein; SP24, putative virion membrane protein of plant and insect virus. The scale bars indicate the number of nucleotides or amino acid substitutions per site.

**Iflavirus:** Only one *Culex* sample (VN4608) had high RPM for Culex iflavi-like virus 4 (CxIFLV-4) (**Table 2**). This virus is classified in the *Iflavirus* genus (family *Iflaviridae*). *Iflavirus* are ISVs found in a variety of insect species all around the world. Some evidence tends to indicate a broader host spectrum for these viruses as illustrated by the king virus and Chequa *iflavirus* which were isolated from a bat (vertebrate) and crustacean (sea arthropod) respectively [49,50]. Although at lower RPM, other *iflaviruses* were detected such as the Sacbrood virus, a honeybee pathogen and two mosquito viruses, the Yongsan iflavvirus 1 and Aedes iflavi-like virus 1. A single CxIFLV-4 genome was assembled from VN4608 sample with a length of 9683 nt including a single ORF coding for a polyprotein which is post translationally cleaved into several structural and functional proteins (**Fig 9A**). The complete genome (PQ220305) was used for phylogenetic assessment with other CxIFLV-4 sequences. The closest relative was a CxIFLV-4 sequence retrieved from Culex in the USA in 2016 with 98,39% nucleotide identities. Other sequences clustering in the same clade included other CxIFLV-4 isolates as well as Aedes iflavi-like virus and iflavirus sp collected from Switzerland, Spain and Belgium, all sharing ∼ 96% nucleotide identities (**Fig 9C**). A second clade was formed by two CxIFLV-4 sequences of lower nucleotide identities (∼80%) with the first clade which might indicate a different specie.

**Negev virus:** We detected several viral species classified in the taxon *Negevirus* such as the Negev virus, Biggie virus and Biggie-like virus in Culex mosquitoes collected between 2005 and 2014 (**Table 2**). The sample 12739CPR3 was particularly rich in *Negeviruse* reads. *Negevirus* are ISVs sharing genetic relatedness to plant viruses of the *Kitaviridae* family and are found in several continents [44]. The assembled Negev virus genome of samples 12739CPR3 (PQ220312) is 9 492 nt in length and harbours three ORFs, the first coding for a large polyprotein with several catalytic domains with methyltransferases and RdRp activities, the second ORF is coding for a glycoprotein while the last ORF product is the putative SP-24 membrane protein (**Fig 9B**). Interestingly, the gene for SP-24 is also present in the second RNA fragment of CxTLV which might indicate a common ancestry between these viruses (**Fig 7**). The RdRp protein (∼500 aa) of the Québec *Negevirus* were used for phylogenetic analysis along with other *Negevirus* classified in the *Sandewavirus* or *Nelorpivirus* sub-clades. The Québec Negev virus strain clustered with other Negev virus from the USA, Italia and Brazil in the *Nelorpivirus* sub-clades which is a sister clade of plant viruses of the *Kitaviridae* family (**Fig 9D**). The two Biggie virus from Québec formed a clade with other Biggie viruses isolates from the USA and Italy in the *Sandewavirus* sub-clade. All the Negev virus strains share high amino acid identities (∼99%) between each other while the Piuria virus diverged (75%) from the other members of the *Nelorpivirus* clade. Moreover, the RdRp sequence of the Québec Biggie virus strain showed ∼ 32% aa identity with the Negev virus strains. This high level of divergence may suggest that Biggie viruses are rather a different specie than a member of a sub-clade in the *Negevirus* taxon. The plant *Kitaviridae* (*Cilevirus* and *Higrevirus*) showed ∼35−39% aa identity with the Negev virus RdRp sequences. We also verified the relatedness of Negev virus and CxTLV via SP-24 phylogeny since both viruses harbour the SP-24 gene and found no significant identities between the two putative SP-24 proteins (25% amino acid identity and 38% query cover). Both SP-24 appears to be different proteins despite sharing a similar domain scaffold (pfam16504).

## Discussion

We detected 60 viral species including three arboviruses, several ISVs and broad host viruses. Surprisingly, half of these viruses are still not thoroughly classified in a specific taxonomic rank such as family, genus or species in the NCBI database. Although there is a significant bias in the number of each mosquito species of this study, we noticed a higher number of viral species found in *Culex pipiens* mosquitoes compared to the other species. Furthermore, we did not observe a trend in the number of viral species found in *Culex pipiens* over time. Interestingly, some ISVs such as CxMV-4, CxFV, AMNV-1, CxTLV, WWV-6, Marma and Biggie viruses are continuously detected in mosquitoes as a function of time despite the presence of a winter season in the province of Québec. Consequently, these ISVs likely rely on vertical transmission to offspring as a mechanism for persistence in nature. The observed viral diversity spectrum between and within mosquito species might reflect different biological and genetic mechanisms in action such as the mosquito innate immune response, virus-host co-evolution (adaptation) and extrinsic factors such as environmental conditions and feeding behaviours to name a few.

We resolved seven arbovirus genomes previously detected by RT-PCR during the transmission season in Québec, Canada: four WNV, two EEEV and one tripartite SSHV. Phylogenetic analysis of two WNV genomic sequences collected in the province of Québec in 2015 revealed their genetic relationship with other Canadian and American sequences of the WN10 genotype which are currently predominant in North America [40]. The WN10 phenotype was recently characterized using *Culex pipiens* as a model [51]. The authors reported increase infectivity and transmissibility of the WNV variant in *Culex pipiens* which was interpreted as a possible adaptative advantage. This finding is consistent with the increased number of WNV sequences belonging the WN10 genotype since 2010 over the formerly dominant WN02 variants.

Two EEEV genomes collected from *Culiseta melanura* in 2016 in the Lanaudière Health unit showed high identity with a Florida EEEV isolate from a sea bird. The Florida state was proposed to be the major geographical source of EEEV in the United States owing to higher genetic diversity and long-term local persistence of strains compared to isolates from northern states such as New York and Massachusetts where frequent re-introduction of southern EEEV strains were observed [52].

A SSHV segmented genome was fully resolved from a pool of *Ochlerotatus stimulans* collected in 2016 in the north-western region of the province of Québec (Fig 1A). The S segment of SSHV shared high identities with North American strains isolated over nearly six decades arguing in favour of a slow evolution process for the nucleocapsid gene. Possible explanation of the divergent SSHV strain isolated in Massachusetts in 1969 might involve local environmental factors contributing to emergence of variants or alternatively the introduction of new variant from a remote geographical location. Although fragment exchange with the related La Crosse virus is theoretically probable in co-infected mosquitoes, there is a lack of field study evidence of such events between those viruses in nature and fragment reassortment is not believed to be a major driving force in establishing genetic variation in California serogroup viruses [53].

We report the complete genome sequences of eight ISVs namely CxMV‑4, WMV‑6, AMNV-1, Marma virus, Negev virus, Tombus-like virus, CxFL, and CxIFLV-4 as well as one dual host vertebrate virus, FLAV. These ISVs had been initially identified in previous metagenomic surveys of Culex mosquitoes [54–56]. Some of these viruses, including CxFL, Negev virus, and AMNV‑1, replicate efficiently in insect cells but fail to propagate in vertebrate cells, a defining phenotypic feature of ISVs [57–59]. Notably, several of these viruses appear to be part of a conserved core virome in *Culex* mosquitoes from Quebec, Canada. Although true core virome definitions are still rare in northern temperate regions, analogous studies in temperate Europe have documented extensive viral diversity and overlapping viral taxa across *Culex* populations, suggesting that a core set of insect‑associated viruses may be widely distributed in similar climates. For example, meta‑transcriptomic analyses of *Culex pipiens* and *Culex torrentium* in northern Europe identified diverse RNA viruses shared across sites and species, indicative of broadly distributed viral taxa in temperate mosquitoes [60].

Single‑mosquito virome studies from Belgium further highlight species‑specific virome patterns and caution against strictly defining a universal core virome at temperate latitudes, even as shared viral components are detected across individuals [55].

Most of the non-arboviral species detected in this study showed significant nucleotide identity and protein sequence conservation with viral isolates reported from all continents which is consistent with the worldwide distribution of *Culex pipiens* in temperate and subtropical regions [61]. The distribution of these viruses is a probable consequence of the globalization of trade and travel facilitating the transportation of arthropod vectors and or viremic hosts to new territories [62]. Interestingly, FLAV are only detected, so far, in the United States and Canada which might indicate a favourable ecosystem established between the avian reservoir, the vectors and the regional climate conditions.

We did not characterize new viral taxa based on sequence identities but we propose a new bipartite virus which we termed Culex Tombus-like virus strain Québec which is composed of two RNA molecules that were originally depicted as two individual viral genomes by another study [48]. One of these viruses termed CxMV-1 was missing a RdRp gene and contained only two ORF coding for a glycoprotein and a membrane protein which is insufficient for replication in the host cells. This is perhaps a limitation of metagenomic assessment studies of mosquito pools which lacks prior viral isolation and propagation [63]. Moreover, the rapid expansion of metagenomic data is not matched by parallel functional studies, limiting our ability to classify novel viral entities within appropriate taxonomic ranks. However, the data provided by functional studies of mosquito interactome continue to accumulate, and the outlook is highly promising for a range of practical applications, particularly in the development of biocontrol agents aimed at reducing arboviral disease transmission [64]. It is important to recall that vector-borne disease accounts for 17% of global infectious disease [65] and there is an urgent need to develop tools and methods to mitigate arbovirus transmission in human populations.

The modulatory property of some ISV in the replication of arboviruses in mosquito host cells and transmission is supported by several *in vitro* and *in vivo* investigations [66–71]. The only insect specific flavivirus found in this study with probable effect on WNV replication is CxFV, a related *flavivirus*. A positive ecological association between WNV and CxFV was found by a study of *Culex* mosquitoes thriving in an endemic focus of WNV transmission in Chicago, USA [72]. However, the inhibition activities presented in the scientific literature is not strong, and the data is inconclusive between studies and varies greatly with mosquito species [71,73]. Interestingly, the superinfection exclusion phenomenon is also described between unrelated viruses. Indeed, the Negev virus, an ISV detected in our study was demonstrated to inhibit the chikungunya virus replication in *Aedes albopictus* cell lines [66].

On the same topic, the bacteria *Wolbachia* sp*.* which we detected in several *Culex pipiens* mosquito is currently used as an authorized biocontrol agent in different countries afflicted by arboviruses [74]. *Wolbachia* sp. are intracellular, Gram-negative endosymbiotic bacteria found in many arthropods and filarial nematodes [33]. Depending on the host species and strain, they can establish either parasitic or mutualistic associations. *Wolbachia sp.* are able to reduce the ability of *Aedes aegypti* mosquitoes to transmit flaviviruses or alphaviruses such as dengue, yellow fever, Zika and Chikungunya viruses [19,20]. Furthermore, a correlation was found between the abundance of *Wolbachia*, the mean temperature and the presence of WNV in *Cx. pipiens/ restuans* collected in Ontario, Canada [75]. It was shown that at higher temperature the abundance of *Wolbachia* species in the microbiome was reduced leading to greater susceptibility to WNV infection in the subsequent generation of *Cx pipiens/restuans* hosts.

Although host expansion of ISVs to vertebrates is conceivable, achieving efficient replication in vertebrate hosts would probably entail fitness trade-offs that compromise replication in mosquito vectors [76]*.* While these constraints likely restrict sustained replication in vertebrates, isolated cases of ISVs detection in vertebrates have been described. For example, a novel *Mivirus*, the Nuomin virus (*Chuviridae*) was isolated from febrile patients in China and its presence confirmed in 54 patients who had a tick-bitten history [77]. This virus was also detected in hard ticks, sheep and cattle in China. Apart from the tick-borne Nuomin virus, no other members of the *Chuviridae* family have so far been reported to cause disease in humans. Although Mesonivirus are considered non-pathogenic in vertebrate hosts, two fatal equine cases related to AMNV-1 infection were reported for the first time in Italy in 2021 [78]. The AMNV-1 was specifically detected by metagenomic and histologic methods in lymph nodes and lung tissues from two horses diagnosed with acute respiratory syndrome. We detected AMNV-1 in three *Culex pipiens* mosquito pools, one from Laval in 2005 and two from Montreal collected in 2014 and 2016. To date there is no AMNV-1 infection cases reported in equids in Canada. A study in Egypt reported the presence of neutralizing antibodies in 8% of the selected human population against the Quaranfil virus (QRFV) [79]. In another study, the experimental intranasal inoculation of QRFV in mice caused neurological disturbance and high mortality [80]. QRFV, like WMV6 detected in this study, is a member of the genus *Quaranjavirus* (family *Orthomyxoviridae*), and neither virus has so far been associated with human disease.

Since hematophagous vectors are frequently in contact with vertebrates, including humans, it is theoretically possible that, over time, certain ISVs could acquire the ability to infect humans, potentially leading to clinical illness or mild, undiagnosed infections [9]. The genetic (viral mutation) and biological processes enabling ISVs to become new vertebrate pathogens are complex [81]. Other biotic or abiotic factors such as vector competence and abundance, human settlement expansion near wildlife habitat and climate change play a central role in creating favourable conditions for the emergence of new arboviruses [82].

The metagenomic sequencing method used in this study was obviously less sensitive (73%) than the classical directed RT-PCR method used in arboviral surveillance programs. As the sequencing technologies become more sensitive and less expensive over time, it will be cost-effective to implement those technologies in arboviral surveillance programs. For example, the monitoring of the spread and extend of new SARS-CoV2 variants was successfully performed through metagenomic sequencing of municipal wastewater [83].

The limitations of this study include the uneven representation of mosquito species, which may introduce bias in the characterization of species-specific core viromes. Although some viruses identified here are likely genuine components of the mosquito microbiota, as supported by findings from similar studies, alternative explanations remain possible. In particular, viral acquisition through plant feeding or mechanical carriages cannot be excluded, especially for viruses known to strictly infect plants. Furthermore, using exclusively arbovirus-positive pools may bias the analysis and limit the ability to comprehensively assess viral diversity, as it restricts observations to already infected samples and may overlook viruses present in uninfected or low-prevalence populations. In addition, the use of pooled mosquitoes rather than individual specimens complicates interpretation, as detected viral sequences may originate from different individuals within the same pool, making it difficult to confidently establish true mixed infections within a single mosquito. This limitation is further exacerbated when pools contain varying numbers of mosquitoes (e.g., from 1 to 50), as differences in pool size can influence viral detection sensitivity and relative abundance estimates, thereby introducing variability that hinders direct comparisons across samples.

In conclusion, we resolved thirty-five complete or segmented viral genomes from medically important mosquito species circulating in Québec, Canada. Our analyses revealed that local mosquito populations harbour a diverse array of viral species sharing significant genetic relatedness with viruses reported from multiple regions worldwide. The data generated in this study contribute to the expanding knowledge of mosquito species and their associated viromes in Québec, a northern region where arbovirus transmission occurs exclusively during the summer months. This knowledge will support field biologists and public health epidemiologists in assessing the risk of human exposure to these viruses and may aid in the early identification of emerging pathogens.

Importantly, this study demonstrates the utility of whole genome sequencing for the surveillance and characterization of arboviruses endemic to the province of Québec. In addition, we identified several ISVs closely associated with medically relevant mosquito vectors. Climate change–driven extensions of transmission seasons may increase exposure of human and animal populations to these viruses. Consequently, the presence of ISVs in vertebrate-seeking vectors could facilitate viral adaptation to new hosts, potentially leading to the emergence of novel pathogens over time. To mitigate the risk of arboviral infections and to monitor potential emerging pathogens, we recommend the implementation of sensitive, continuous entomological surveillance programs incorporating metagenomic sequencing. Comprehensive field data collection will also be essential to support and initiate urgently needed fundamental and applied research efforts aimed at protecting human and wildlife populations in Canada.

## Supporting information

S1 Table Detection and abundance of bacterial taxon in three mosquito species from 2005 to 2016 expressed as reads per million (RPM). (DOCX)

S2 Table Detection and abundance of eukaryota taxon in three mosquito species from 2005 to 2016 expressed as reads per million (RPM). (DOCX)

S3 Table Sequencing metrics for the 35 complete or near complete viral genomes/segments. (XLSX)
