# Supplementary material for: RNA metagenomic profiling of mosquito viromes associated with Vector-Borne diseases in Quebec, Canada
Source: PLoS One. 2026 Jun 5;21(6):e0350663. doi: 10.1371/journal.pone.0350663 (PMC13240903; doi:10.1371/journal.pone.0350663)
Supplement: S1 Table — (DOCX) [file pone.0350663.s001.docx]

**S1 Table.** Detection and abundance of bacterial taxon in three mosquito species from 2005 to 2016 expressed as reads per million (RPM).

| Taxa | VN6831CPR1 | VN6387CPR1R | VN4608CPR1 | 12739CPR3 | VN6325CPR1 | VN4593CPR1 | VN6477CPR1R | VN6307CPR1R | 13386CPR1 | 13896CPR1 | VN6800CPR1 | 12867CPR2 | VN6711SMG1 | VN6966SMG1 | VN6827MEL1 | VN7173MEL1 |
| --- | --- | --- | --- | --- | --- | --- | --- | --- | --- | --- | --- | --- | --- | --- | --- | --- |
| Acetobacter | 0 | 0 | 0 | 0 | 0 | 0 | 0 | 0 | 0 | 0 | 0 | 0 | 0 | 0 | 30 | 51 |
| Acidovorax | 90 | 28 | 0 | 0 | 0 | 0 | 0 | 0 | 0 | 0 | 0 | 0 | 0 | 0 | 0 | 0 |
| Acinetobacter | 0 | 0 | 0 | 0 | 0 | 0 | 0 | 0 | 0 | 0 | 0 | 146 | 0 | 0 | 34 | 0 |
| Aeromonas | 0 | 0 | 0 | 0 | 0 | 0 | 0 | 0 | 0 | 151 | 0 | 0 | 0 | 0 | 0 | 0 |
| Anaplasma | 70 | 0 | 0 | 0 | 0 | 0 | 0 | 0 | 0 | 0 | 0 | 0 | 0 | 0 | 0 | 0 |
| Asaia | 0 | 0 | 0 | 0 | 0 | 0 | 0 | 0 | 101 | 0 | 0 | 0 | 0 | 90 | 187 | 1528 |
| Atlantibacter | 0 | 0 | 0 | 0 | 0 | 0 | 0 | 0 | 0 | 0 | 0 | 0 | 0 | 0 | 53 | 0 |
| Avibacterium | 0 | 0 | 0 | 0 | 0 | 0 | 0 | 0 | 0 | 0 | 0 | 0 | 0 | 0 | 46 | 0 |
| Bacillus | 0 | 0 | 0 | 0 | 0 | 0 | 0 | 0 | 0 | 0 | 0 | 0 | 57 | 0 | 0 | 0 |
| Blattabacterium | 709 | 0 | 0 | 0 | 0 | 0 | 0 | 0 | 0 | 0 | 0 | 0 | 0 | 0 | 0 | 0 |
| Borrelia | 76 | 0 | 0 | 0 | 0 | 0 | 0 | 0 | 0 | 0 | 0 | 98 | 0 | 0 | 830 | 407 |
| Borreliella | 0 | 0 | 0 | 0 | 0 | 0 | 0 | 0 | 0 | 0 | 0 | 0 | 0 | 0 | 1256 | 0 |
| Buchnera | 533 | 101 | 0 | 0 | 0 | 0 | 0 | 0 | 0 | 0 | 0 | 0 | 0 | 0 | 0 | 0 |
| Candidatus Azobacteroides | 31 | 0 | 0 | 0 | 0 | 0 | 0 | 0 | 0 | 0 | 0 | 0 | 0 | 0 | 0 | 0 |
| Candidatus Blochmannia | 83 | 0 | 0 | 0 | 0 | 0 | 0 | 0 | 0 | 0 | 0 | 0 | 0 | 0 | 0 | 0 |
| Candidatus Riesia | 30 | 0 | 0 | 0 | 0 | 0 | 0 | 0 | 0 | 0 | 0 | 0 | 0 | 0 | 0 | 0 |
| Candidatus Sulcia | 86 | 0 | 0 | 0 | 0 | 0 | 0 | 0 | 0 | 0 | 0 | 0 | 0 | 0 | 0 | 0 |
| Candidatus Tachikawaea | 1688 | 0 | 0 | 0 | 0 | 0 | 0 | 0 | 0 | 0 | 0 | 0 | 0 | 0 | 0 | 0 |
| Candidatus Tenderia | 0 | 0 | 0 | 0 | 0 | 0 | 189 | 0 | 0 | 0 | 0 | 0 | 0 | 0 | 0 | 0 |
| Candidatus Thioglobus | 0 | 0 | 0 | 0 | 0 | 0 | 0 | 0 | 0 | 0 | 0 | 0 | 0 | 0 | 43 | 0 |
| Cedecea | 0 | 0 | 0 | 0 | 0 | 0 | 0 | 0 | 0 | 0 | 0 | 0 | 204 | 0 | 46 | 0 |
| Citrobacter | 57 | 0 | 79 | 0 | 0 | 0 | 0 | 0 | 0 | 0 | 0 | 0 | 0 | 0 | 72 | 0 |
| Desulfovibrio | 140 | 0 | 0 | 0 | 0 | 0 | 0 | 0 | 0 | 0 | 0 | 0 | 0 | 0 | 0 | 44 |
| Desulfuromonas | 0 | 0 | 0 | 0 | 0 | 0 | 0 | 0 | 0 | 0 | 0 | 35 | 0 | 0 | 0 | 0 |
| Dialister | 0 | 0 | 0 | 0 | 0 | 0 | 0 | 0 | 0 | 0 | 0 | 0 | 30 | 0 | 0 | 0 |
| Dickeya | 1320 | 0 | 0 | 0 | 0 | 0 | 0 | 0 | 0 | 0 | 0 | 0 | 0 | 0 | 0 | 0 |
| Edwardsiella | 0 | 0 | 0 | 0 | 0 | 0 | 0 | 0 | 0 | 0 | 0 | 0 | 0 | 0 | 26 | 0 |
| Ehrlichia | 160 | 0 | 59 | 155 | 56 | 69 | 32 | 0 | 0 | 0 | 0 | 357 | 0 | 0 | 0 | 0 |
| Empedobacter | 31 | 0 | 0 | 0 | 0 | 0 | 0 | 0 | 0 | 0 | 0 | 0 | 0 | 0 | 0 | 0 |
| Enterobacter | 143 | 0 | 38 | 0 | 0 | 0 | 0 | 0 | 0 | 0 | 0 | 0 | 0 | 0 | 47 | 0 |
| Entomoplasma | 0 | 0 | 0 | 0 | 0 | 0 | 0 | 0 | 0 | 0 | 0 | 0 | 39 | 0 | 0 | 0 |
| Erwinia | 30 | 210 | 0 | 74 | 0 | 0 | 423 | 42 | 0 | 0 | 0 | 87 | 0 | 26 | 0 | 181 |
| Escherichia | 177 | 36 | 54 | 0 | 0 | 0 | 0 | 0 | 115 | 0 | 0 | 0 | 31 | 0 | 333 | 37 |
| Frischella | 0 | 0 | 0 | 0 | 0 | 0 | 0 | 0 | 0 | 0 | 0 | 0 | 0 | 0 | 108 | 0 |
| Fusobacterium | 0 | 0 | 0 | 0 | 0 | 0 | 0 | 0 | 0 | 0 | 0 | 0 | 0 | 0 | 178 | 0 |
| Gibbsiella | 0 | 0 | 0 | 0 | 0 | 0 | 0 | 0 | 0 | 0 | 0 | 0 | 81 | 0 | 0 | 0 |
| Gilliamella | 34 | 0 | 0 | 0 | 0 | 0 | 0 | 0 | 0 | 0 | 0 | 0 | 0 | 0 | 1168 | 125 |
| Halioglobus | 0 | 0 | 0 | 0 | 0 | 0 | 0 | 0 | 0 | 0 | 0 | 76 | 0 | 0 | 0 | 0 |
| Klebsiella | 160 | 121 | 0 | 0 | 0 | 0 | 0 | 0 | 0 | 0 | 0 | 0 | 32 | 0 | 332 | 32 |
| Komagataeibacter | 0 | 0 | 0 | 0 | 0 | 0 | 0 | 0 | 0 | 0 | 0 | 0 | 0 | 0 | 0 | 48 |
| Kozakia | 0 | 0 | 0 | 0 | 0 | 0 | 0 | 0 | 0 | 0 | 0 | 0 | 0 | 0 | 0 | 51 |
| Kurthia | 0 | 0 | 0 | 0 | 0 | 0 | 0 | 0 | 0 | 0 | 0 | 0 | 0 | 0 | 0 | 33 |
| Lactiplantibacillus | 0 | 0 | 0 | 0 | 0 | 0 | 0 | 0 | 0 | 0 | 0 | 88 | 0 | 0 | 0 | 0 |
| Lactococcus | 0 | 0 | 0 | 0 | 0 | 72 | 0 | 0 | 0 | 0 | 0 | 0 | 0 | 0 | 0 | 0 |
| Leclercia | 26 | 185 | 0 | 0 | 0 | 0 | 0 | 0 | 0 | 0 | 0 | 0 | 0 | 0 | 0 | 0 |
| Lelliottia | 108 | 0 | 0 | 0 | 0 | 0 | 0 | 0 | 0 | 0 | 0 | 0 | 0 | 0 | 0 | 0 |
| Leuconostoc | 0 | 0 | 0 | 0 | 0 | 0 | 0 | 0 | 0 | 0 | 0 | 0 | 0 | 0 | 0 | 32 |
| Marinobacterium | 0 | 0 | 0 | 0 | 0 | 0 | 0 | 0 | 0 | 0 | 0 | 0 | 0 | 0 | 0 | 50 |
| Mesoplasma | 0 | 0 | 0 | 0 | 0 | 0 | 0 | 0 | 0 | 0 | 0 | 0 | 29 | 0 | 0 | 0 |
| Mycobacterium | 0 | 0 | 0 | 0 | 0 | 0 | 0 | 0 | 0 | 0 | 0 | 0 | 0 | 0 | 32 | 0 |
| Mycoplasma | 26 | 0 | 0 | 0 | 0 | 0 | 0 | 0 | 0 | 0 | 0 | 437 | 115 | 0 | 0 | 27 |
| Neoasaia | 0 | 0 | 0 | 0 | 0 | 0 | 0 | 0 | 0 | 0 | 0 | 0 | 0 | 0 | 0 | 54 |
| Oceanispirochaeta | 1511 | 0 | 30 | 55 | 0 | 28 | 0 | 0 | 0 | 0 | 0 | 280 | 0 | 0 | 860 | 1117 |
| Orbus | 0 | 0 | 0 | 0 | 0 | 0 | 0 | 0 | 0 | 0 | 0 | 0 | 0 | 0 | 168 | 56 |
| Pantoea | 231 | 85 | 0 | 0 | 30 | 0 | 0 | 545 | 0 | 0 | 0 | 125 | 0 | 0 | 31 | 170 |
| Pluralibacter | 30 | 0 | 29 | 0 | 0 | 0 | 0 | 0 | 0 | 0 | 0 | 0 | 0 | 0 | 0 | 0 |
| Providencia | 0 | 0 | 0 | 0 | 0 | 0 | 0 | 0 | 0 | 0 | 0 | 0 | 0 | 0 | 32 | 0 |
| Pseudomonas | 0 | 33 | 0 | 0 | 0 | 0 | 33 | 0 | 0 | 0 | 0 | 0 | 0 | 0 | 0 | 709 |
| Rahnella | 110 | 0 | 0 | 0 | 0 | 0 | 0 | 0 | 0 | 0 | 0 | 119 | 0 | 0 | 84 | 81 |
| Raoultella | 0 | 0 | 34 | 0 | 0 | 0 | 0 | 0 | 0 | 0 | 0 | 0 | 0 | 0 | 169 | 0 |
| Rickettsiella | 0 | 387 | 0 | 0 | 0 | 0 | 0 | 0 | 0 | 0 | 0 | 0 | 0 | 0 | 0 | 0 |
| Salmonella | 0 | 0 | 0 | 0 | 0 | 0 | 0 | 0 | 0 | 0 | 0 | 0 | 0 | 0 | 40 | 0 |
| Sediminispirochaeta | 36 | 34 | 0 | 0 | 0 | 0 | 0 | 0 | 0 | 0 | 0 | 0 | 0 | 0 | 59 | 40 |
| Serratia | 52 | 327 | 25 | 0 | 0 | 0 | 0 | 0 | 0 | 0 | 0 | 0 | 30 | 0 | 53 | 0 |
| Sodalis | 57 | 0 | 0 | 0 | 0 | 0 | 0 | 0 | 0 | 0 | 0 | 0 | 0 | 0 | 0 | 0 |
| Spirochaeta | 0 | 0 | 0 | 0 | 0 | 0 | 0 | 0 | 0 | 0 | 0 | 0 | 0 | 0 | 86 | 0 |
| Spironema | 109 | 331 | 0 | 80 | 77 | 65 | 256 | 0 | 0 | 0 | 0 | 359 | 0 | 0 | 442 | 808 |
| Spiroplasma | 0 | 0 | 0 | 30 | 0 | 0 | 0 | 0 | 0 | 0 | 0 | 0 | 45332 | 15745 | 0 | 0 |
| Staphylococcus | 0 | 0 | 0 | 0 | 0 | 0 | 0 | 0 | 0 | 0 | 0 | 0 | 69 | 0 | 0 | 0 |
| Streptococcus | 0 | 0 | 0 | 0 | 0 | 0 | 0 | 0 | 27 | 0 | 0 | 0 | 0 | 0 | 0 | 0 |
| Sulfurimonas | 0 | 0 | 0 | 0 | 0 | 0 | 0 | 0 | 0 | 0 | 0 | 0 | 0 | 0 | 0 | 36 |
| Swaminathania | 0 | 0 | 0 | 0 | 0 | 0 | 0 | 0 | 47 | 0 | 0 | 0 | 0 | 0 | 0 | 0 |
| Tatumella | 0 | 0 | 0 | 0 | 0 | 0 | 0 | 0 | 0 | 0 | 0 | 0 | 0 | 0 | 0 | 496 |
| Thiospirochaeta | 370 | 0 | 0 | 0 | 147 | 75 | 0 | 0 | 0 | 0 | 0 | 1613 | 0 | 0 | 1610 | 1027 |
| Treponema | 292 | 0 | 50 | 38 | 0 | 0 | 0 | 0 | 0 | 0 | 0 | 528 | 0 | 0 | 268 | 120 |
| Turneriella | 0 | 0 | 0 | 0 | 0 | 0 | 0 | 0 | 0 | 0 | 0 | 0 | 0 | 0 | 26 | 0 |
| Ureaplasma | 0 | 0 | 0 | 0 | 0 | 0 | 0 | 0 | 0 | 0 | 0 | 0 | 84 | 0 | 0 | 0 |
| Vibrio | 33 | 0 | 0 | 0 | 0 | 0 | 0 | 0 | 0 | 0 | 0 | 0 | 0 | 0 | 0 | 0 |
| Wolbachia | 70295 | 6110 | 22318 | 21846 | 29641 | 20961 | 10942 | 6242 | 10561 | 699 | 0 | 52381 | 0 | 0 | 0 | 0 |
| Xenorhabdus | 64 | 0 | 0 | 0 | 0 | 0 | 0 | 0 | 0 | 0 | 0 | 0 | 0 | 0 | 0 | 0 |
| Yersinia | 0 | 0 | 0 | 0 | 0 | 0 | 0 | 0 | 0 | 0 | 0 | 0 | 0 | 0 | 52 | 0 |
| Zymobacter | 0 | 0 | 0 | 0 | 0 | 174 | 112 | 0 | 0 | 0 | 0 | 0 | 0 | 0 | 0 | 0 |

82 genera; Metric: NT.rpm; 3 Filters Applied: Categories:Bacteria; Thresholds:NT rPM >= 25; Read Specificity:"All" ; Background: "None"
