# Supplementary material for: RNA metagenomic profiling of mosquito viromes associated with Vector-Borne diseases in Quebec, Canada
Source: PLoS One. 2026 Jun 5;21(6):e0350663. doi: 10.1371/journal.pone.0350663 (PMC13240903; doi:10.1371/journal.pone.0350663)
Supplement: S2 Table — (DOCX) [file pone.0350663.s002.docx]

**S2 Table.** Detection and abundance of Eukaryota taxon in three mosquito species from 2005 to 2016 expressed as reads per million (RPM).

| Taxa | VN6831CPR1 | VN4608CPR1 | 12867CPR2 | 12739CPR3 | VN4593CPR1 | 13386CPR1 | VN6387CPR1R | VN6477CPR1R | 13896CPR1 | VN6307CPR1R | VN6800CPR1 | VN6325CPR1 | VN6711SMG1 | VN6966SMG1 | VN7173MEL1 | VN6827MEL1 |
| --- | --- | --- | --- | --- | --- | --- | --- | --- | --- | --- | --- | --- | --- | --- | --- | --- |
| Aedes | 34 | 55 | 0 | 0 | 0 | 0 | 0 | 0 | 0 | 0 | 0 | 0 | 1743 | 3180 | 239 | 179 |
| Amblyospora | 410 | 1469 | 465 | 0 | 0 | 0 | 0 | 0 | 0 | 0 | 0 | 0 | 14518 | 1646 | 269 | 0 |
| Anncaliia | 314 | 347 | 71 | 0 | 0 | 0 | 0 | 0 | 0 | 0 | 0 | 0 | 1742 | 65 | 0 | 0 |
| Attaphila | 0 | 1283 | 0 | 0 | 0 | 0 | 0 | 0 | 0 | 0 | 0 | 0 | 132 | 0 | 0 | 0 |
| Babesia | 452 | 0 | 0 | 0 | 0 | 0 | 0 | 0 | 0 | 0 | 0 | 0 | 0 | 0 | 0 | 0 |
| Bodo | 0 | 0 | 0 | 0 | 0 | 32 | 0 | 0 | 0 | 0 | 0 | 0 | 0 | 0 | 0 | 0 |
| Chironomus | 0 | 792 | 0 | 0 | 85 | 0 | 27 | 0 | 0 | 0 | 0 | 0 | 0 | 0 | 0 | 0 |
| Cladosporium | 0 | 86 | 0 | 0 | 0 | 0 | 0 | 62 | 0 | 0 | 0 | 0 | 0 | 0 | 0 | 0 |
| Claviceps | 0 | 0 | 0 | 0 | 0 | 0 | 0 | 432 | 0 | 0 | 0 | 0 | 0 | 0 | 0 | 0 |
| Contarinia | 532 | 0 | 0 | 0 | 0 | 0 | 177 | 0 | 0 | 0 | 0 | 0 | 0 | 0 | 0 | 0 |
| Coquillettidia | 0 | 0 | 0 | 0 | 0 | 0 | 0 | 0 | 0 | 0 | 0 | 0 | 0 | 0 | 659 | 663 |
| Crithidia | 0 | 0 | 0 | 0 | 0 | 0 | 0 | 0 | 0 | 0 | 0 | 0 | 0 | 358 | 485 | 1589 |
| Cryobiotus | 958 | 0 | 0 | 0 | 0 | 0 | 0 | 0 | 0 | 0 | 0 | 0 | 712 | 0 | 0 | 0 |
| Culex | 539 | 106 | 139 | 36 | 132 | 54 | 63 | 148 | 68 | 212 | 570 | 0 | 35 | 44 | 174 | 551 |
| Culicospora | 1880 | 2728 | 591 | 0 | 0 | 0 | 0 | 0 | 0 | 0 | 0 | 0 | 0 | 0 | 0 | 0 |
| Culiseta | 0 | 0 | 0 | 0 | 0 | 0 | 0 | 0 | 0 | 0 | 0 | 0 | 0 | 0 | 15097 | 10597 |
| Dirofilaria | 51 | 0 | 0 | 0 | 28 | 0 | 0 | 0 | 0 | 0 | 0 | 0 | 26 | 0 | 0 | 0 |
| Encephalitozoon | 0 | 71 | 0 | 0 | 0 | 0 | 0 | 0 | 0 | 0 | 0 | 0 | 1579 | 62 | 0 | 0 |
| Endoreticulatus | 1734 | 713 | 73 | 27 | 0 | 0 | 0 | 0 | 0 | 0 | 0 | 0 | 4542 | 263 | 0 | 0 |
| Entomophaga | 101 | 0 | 0 | 0 | 0 | 0 | 0 | 0 | 169 | 0 | 0 | 0 | 0 | 0 | 0 | 0 |
| Entomophthora | 0 | 0 | 0 | 0 | 0 | 0 | 0 | 0 | 54 | 0 | 0 | 0 | 0 | 0 | 0 | 0 |
| Epichloe | 0 | 0 | 0 | 0 | 0 | 0 | 0 | 70 | 0 | 0 | 0 | 0 | 0 | 0 | 0 | 0 |
| Eryniopsis | 0 | 0 | 0 | 0 | 0 | 0 | 0 | 0 | 30 | 0 | 0 | 0 | 0 | 0 | 0 | 0 |
| Eurydema | 0 | 0 | 0 | 0 | 0 | 0 | 0 | 0 | 0 | 0 | 0 | 0 | 1722 | 0 | 0 | 0 |
| Fusarium | 0 | 0 | 0 | 0 | 0 | 0 | 0 | 234 | 0 | 0 | 0 | 0 | 0 | 0 | 0 | 0 |
| Glugoides | 127 | 94 | 0 | 29 | 0 | 0 | 0 | 0 | 0 | 0 | 0 | 0 | 315 | 0 | 0 | 0 |
| Hyalinocysta | 0 | 0 | 0 | 0 | 0 | 0 | 0 | 0 | 0 | 0 | 0 | 0 | 229 | 0 | 5699 | 0 |
| Lasioptera | 35 | 0 | 0 | 0 | 0 | 0 | 152 | 0 | 0 | 0 | 0 | 0 | 0 | 0 | 0 | 0 |
| Leishmania | 0 | 0 | 64 | 28 | 42 | 250 | 330 | 0 | 0 | 0 | 0 | 0 | 0 | 49 | 66 | 202 |
| Leptoglossus | 0 | 0 | 0 | 0 | 0 | 0 | 0 | 0 | 0 | 0 | 0 | 0 | 0 | 78 | 0 | 0 |
| Leptomonas | 0 | 0 | 0 | 0 | 0 | 0 | 0 | 0 | 0 | 0 | 0 | 0 | 0 | 0 | 0 | 207 |
| Metarhizium | 0 | 0 | 0 | 0 | 0 | 0 | 0 | 26 | 0 | 0 | 0 | 0 | 0 | 0 | 0 | 0 |
| Microsporidium | 290 | 1416 | 0 | 194 | 0 | 0 | 0 | 0 | 0 | 0 | 0 | 0 | 3109 | 0 | 367 | 0 |
| Mycodiplosis | 151 | 0 | 0 | 0 | 0 | 0 | 245 | 0 | 0 | 0 | 0 | 0 | 0 | 0 | 0 | 0 |
| Ochlerotatus | 0 | 0 | 0 | 0 | 0 | 0 | 0 | 0 | 0 | 0 | 0 | 0 | 2548 | 4999 | 1444 | 1108 |
| Paraepiseptum | 0 | 0 | 0 | 0 | 0 | 0 | 0 | 0 | 0 | 0 | 0 | 0 | 0 | 0 | 439 | 0 |
| Paratrypanosoma | 0 | 0 | 247 | 54 | 286 | 354 | 642 | 0 | 31 | 0 | 0 | 0 | 0 | 0 | 0 | 0 |
| Plasmodium | 33 | 123 | 0 | 35 | 156 | 0 | 0 | 0 | 0 | 57 | 0 | 0 | 0 | 0 | 0 | 55 |
| Ramazzottius | 0 | 673 | 101 | 0 | 0 | 0 | 0 | 0 | 0 | 0 | 0 | 0 | 0 | 255 | 0 | 0 |
| Riptortus | 50 | 0 | 29 | 108 | 0 | 84 | 0 | 0 | 0 | 0 | 0 | 0 | 0 | 0 | 0 | 0 |
| Sciophila | 0 | 0 | 0 | 0 | 0 | 0 | 47 | 0 | 0 | 0 | 0 | 0 | 0 | 0 | 0 | 0 |
| Storthyngura | 0 | 0 | 0 | 0 | 0 | 0 | 0 | 0 | 0 | 0 | 0 | 0 | 4691 | 0 | 0 | 0 |
| Timema | 41 | 35 | 0 | 0 | 0 | 0 | 0 | 0 | 0 | 0 | 0 | 52 | 0 | 0 | 0 | 0 |
| Trypanosoma | 0 | 561 | 426 | 124 | 339 | 590 | 154 | 0 | 0 | 0 | 0 | 0 | 896 | 0 | 1462 | 1899 |
| Uranotaenia | 0 | 0 | 0 | 0 | 0 | 0 | 0 | 0 | 0 | 0 | 0 | 0 | 0 | 0 | 248 | 152 |
| Vavraia | 108 | 101 | 31 | 0 | 0 | 0 | 0 | 0 | 0 | 0 | 0 | 0 | 18945 | 697 | 0 | 0 |
| Verticillium | 0 | 0 | 0 | 0 | 0 | 0 | 0 | 163 | 0 | 0 | 0 | 0 | 0 | 0 | 0 | 0 |
| Vittaforma | 1523 | 84 | 0 | 140 | 0 | 0 | 0 | 0 | 0 | 0 | 0 | 0 | 210 | 0 | 0 | 0 |
| Zelonia | 0 | 0 | 136 | 0 | 0 | 0 | 467 | 0 | 0 | 0 | 0 | 0 | 0 | 0 | 0 | 0 |

49 genera; Metric: NT.rpm; 3 Filters Applied: Categories:Eukaryorta; Thresholds:NT rPM >= 25; Read Specificity:"All" ; Background: "None"

Mosquitoes; Microsporidia insect parasites.
